# Dual functional properties of a probiotic biofilm-decorated bone substitute to combat infection and promote osteoimmunomodulation

**DOI:** 10.1016/j.bioactmat.2025.11.019

**Published:** 2025-11-18

**Authors:** Shiyuan Song, Wen Zhang, Hongmei Zhuang, Wei Wei, Shuyu Cheng, Dan Qiao, Yin Xiao, Yangheng Zhang, Fuhua Yan

**Affiliations:** a Nanjing Stomatological Hospital, Affiliated Hospital of Medical School, Institute of Stomology, Nanjing University, Nanjing, 210008, China; b Qinghai University Affiliated Hospital, Xining, 810000, China; c School of Medicine and Dentistry & Institute for Biomedicine and Glycomics, Griffith University, QLD, 4222, Australia

**Keywords:** *Akkermansia muciniphila*, Anti-bacterial adhesion, Osteoinductivity, IL-10, PI3K/AKT, Osteoimmunology, Bone

## Abstract

Oral and maxillofacial bone defects are prevalent and challenging to treat, often leading to significant complications such as infection and impaired healing. Enhancing the antibacterial and osteoimmunomodulatory properties of bone graft materials represents a promising approach to improve regenerative outcomes. In this study, we developed a hydroxyapatite scaffold coated with a biofilm of the probiotic *Akkermansia muciniphila* (*Akk*-HA), engineered to simultaneously combat infection and modulate the immune environment. *Akk*-HA exhibited vigorous anti-adhesive activity against pathogenic bacteria and attenuated inflammatory responses by suppressing proinflammatory cytokine secretion while promoting the release of proregenerative mediators from macrophages. Mechanistic studies revealed that *Akk*-HA activated the PI3K/AKT signalling pathway, leading to the upregulation of interleukin-10 (IL-10), in turn enhancing the osteogenic differentiation of periodontal ligament cells (PDLCs). In a murine model of infected periodontal bone defects, *Akk*-HA demonstrated significant antibacterial and immunomodulatory effects, resulting in markedly improved bone regeneration. These findings highlight the therapeutic potential of probiotic-functionalized bone grafts as a dual-action strategy for managing infected bone defects in the oral and maxillofacial regions.

## Highlights

• *Akk*-HA exhibits robust anti-bacterial adhesion properties.
• *Akk-HA* reprograms macrophages into a pro-regenerative phenotype and promotes osteogenesis in PDLCs and BMSCs.
• *Akk-*HA enhances bone regeneration by suppressing infection and modulating immunity.

## Graphical abstract

**Figure:**
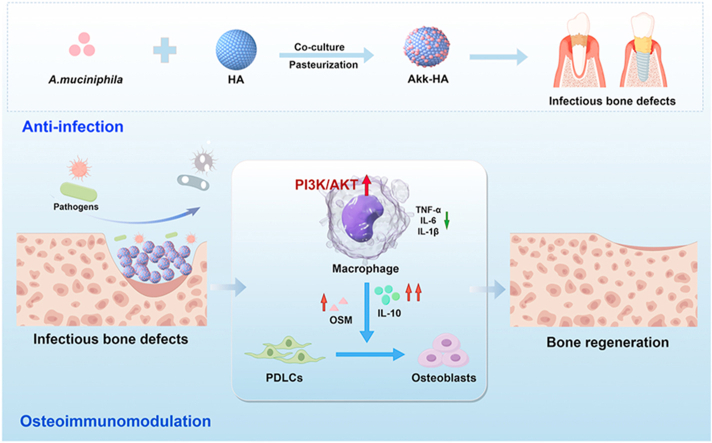
Schematic illustration of the use of *Akk*-HA to prevent infection and enhance osteoimmunomodulation.

## 1 Introduction

Oral and maxillofacial bone defects pose significant clinical challenges, adversely impacting craniofacial integrity, occlusal function, and patient quality of life. These defects often occur in complex anatomical sites that are susceptible to bacterial infection [1]. Infected bone defects, such as defects caused by periodontitis, peri-implantitis, periapical bone lesions and osteomyelitis of the jaw, are characterized by bacterial infection and bone loss [2]. Effective management of these conditions necessitates both local infection control and the promotion of bone healing to achieve successful restoration.

Bone replacement materials represent a key component of repair strategies. Current bone replacement materials include autografts, allografts, xenografts, and synthetic substitutes such as hydroxyapatite (HA) and calcium phosphate cements [3]. Despite their widespread use, these biomaterials face two core issues in current clinical applications: susceptibility to pathogen colonization at the interface and a lack of inherent immunomodulatory capacity [4,5]. Pathogen colonization on biomaterial surfaces can outcompete host cell integration, inducing local immunosuppression that facilitates persistent biofilm infections. These biofilm-associated infections frequently exhibit multidrug resistance, rendering conventional antibiotic therapies ineffective [6]. Emerging evidence in bone immunology confirms the dynamic crosstalk between the immune and skeletal systems. Traditional bioinert materials, lacking immunomodulatory activity, fail to evoke the immune microenvironment remodelling that is essential for bone regeneration [5]. Consequently, developing bone replacement materials with both anti-bacterial adhesion and osteoimmunomodulatory properties is critical for the effective repair of bone defects with a high risk of infection.

The immunomodulatory effects of intestinal probiotics have been well established in recent years [7,8]. *Akkermansia muciniphila* (*A. muciniphila, Akk*), a key member probiotic species colonizing the intestinal mucosal layer, typically constitutes 4 % of the total gut microbiota [9]. This mucus-degrading bacterium plays critical metabolic roles by releasing short-chain fatty acids (SCFAs), which maintain intestinal epithelial homeostasis and provide metabolic substrates for symbiotic bacteria [10]. Its competitive growth underlies its ability to inhibit pathogenic species. In addition to metabolic functions, *A. muciniphila* directly modulates various immune cells, including dendritic cells, T cells and macrophages [11]. Notably, under inflammatory conditions, *A. muciniphila* can promote macrophage polarization from the inflammatory M1 phenotype to the anti-inflammatory M2 phenotype [12]. Furthermore, the daily administration of live *A. muciniphila* by gavage was found to reduce periodontal inflammation and tissue destruction in experimental periodontitis models [13], highlighting the potential therapeutic value of this treatment for oral and maxillofacial bone defects.

While live probiotics are generally considered safe, their clinical application has inherent potential risks, including systemic infection via tissue invasion, antibiotic resistance, and disruption of the gut microbial composition [[14], [15], [16]]. Inactivated probiotics and microbial extracts, which serve as alternative forms of probiotic delivery, are attracting increasing attention because of their enhanced stability and improved safety profiles [17]. Pasteurized *A. muciniphila* retains its structural integrity and biological functions [18]. It retains the ability to regulate lipid metabolism, maintain immune system homeostasis, and modulate the gut microbiota composition, enhancing overall host health [19]. On this basis, we hypothesized that clinically available bone replacement materials (such as HA) modified with pasteurized *A. muciniphila* could effectively resist pathogenic bacterial adhesion and prevent infection while simultaneously modulating macrophage function to establish a regenerative immune microenvironment, thereby promoting the repair of oral and maxillofacial bone defects.

**Scheme 1:**
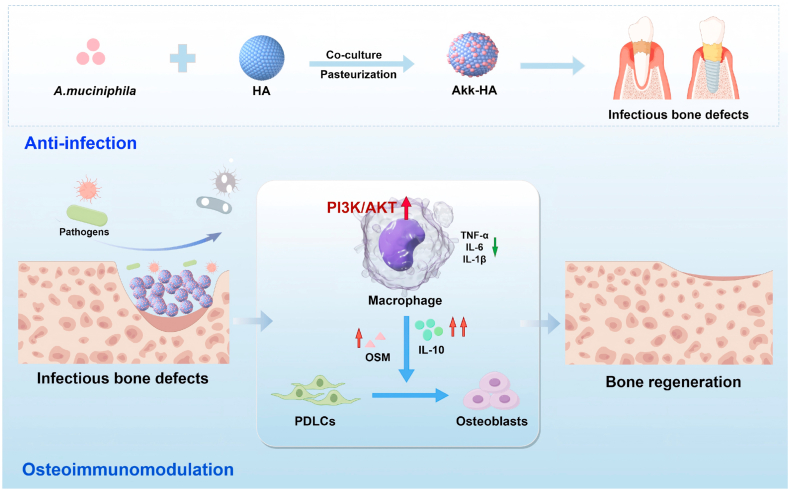
Schematic illustration of the use of *Akk*-HA to prevent infection and enhance osteoimmunomodulation. *Akk*-HA can effectively alleviate pathogen colonization, reduce inflammation and promote bone regeneration, providing a convenient, biocompatible and efficient treatment strategy for treating maxillofacial and other infected bone defects.

Herein, we aimed to develop a dual-function biomaterial that HA functionalized with pasteurized *A. muciniphila* (*Akk*-HA) to overcome the challenges associated with the treatment of oral and maxillofacial bone defects. Compared with other probiotic coatings used to modify bone substitutes, the *A. muciniphila* biofilm modification achieved a cascade of regulatory effects on infection defence, immune homeostasis, and bone regeneration. It conferred potent anti-adhesion activity against pathogens, physically blocking colonization while maintaining biocompatibility. In addition to suppressing inflammation, *Akk*-HA directly reprogrammed macrophages to secrete osteoinductive cytokines via PI3K/AKT-mediated signalling, which activated the osteogenic differentiation of progenitor cells. Crucially, immobilization enabled sustained local biofilm retention, achieving persistent immunomodulation without systemic dissemination, which is not possible with free probiotics. Finally, the dual anti-bacterial adhesion and osteoimmunomodulatory effects of *Akk*-HA were further elucidated *in vivo*. As illustrated in Scheme 1, this approach effectively reduced pathogen colonization, alleviated inflammation, and promoted bone regeneration, providing a convenient, biocompatible, and efficient treatment strategy for treating maxillofacial and other infected bone defects.

## 2 Materials and methods

### 2.1 Fabrication and characterization of *Akk*-HA

#### 2.1.1 Fabrication of Akk-HA

HA microspheres with a diameter of 50 μm were purchased from EPRUI Biotech Co., Ltd. (Shanghai, China). HA at a concentration of 20 mg/mL and *A. muciniphila* (OD600 = 1) were cocultured for 3 days under anaerobic conditions to promote biofilm formation of *A. muciniphila* on the HA surface. Unattached *A. muciniphila* was removed by repeated centrifugation and washing with PBS until bacteria could not be detected in the supernatant. The detailed culture protocols for *A. muciniphila* are described in the Supporting Information. Then, *A. muciniphila* was pasteurized in a temperature-controlled water bath at 70 °C for 30 min. Prepared *Akk*-HA was maintained in a sterile container at 4 °C for subsequent assays. For SYTO 9/PI staining in bacterial adhesion assays and mechanical property assessments, *A. muciniphila* was immobilized on 8-mm-diameter HA discs using the same method to facilitate observation and testing.

#### 2.1.2 Characterization of Akk-HA

Scanning electron microscopy (SEM, Hitachi, Tokyo, Japan) was employed to characterize *A. muciniphila* biofilm formation on HA surfaces and to perform elemental mapping of both *Akk*-HA and HA. For fluorescence staining, the samples were stained with Concanavalin A (Con A) tetramethylrhodamine conjugate (546 nm)/4′,6-diamidino-2-phenylindole (DAPI) (488 nm) and SYTO 9 (500 nm)/PI (635 nm) and then imaged by confocal microscopy (Nikon, Tokyo, Japan). Quantitative analysis of *A. muciniphila* on the HA surface was performed using quantitative polymerase chain reaction (qPCR). The mechanical properties, elemental composition, surface wettability (contact angle), stability, and degradability of *Akk*-HA were systematically characterized through nanoindentation, energy-dispersive X-ray spectroscopy (EDS), static water contact angle measurements, buffer flow assays, long-term culture stability tests, and enzymatic degradation assays. Detailed protocols for bacterial quantification and other methods for the characterization of *Akk*-HA are provided in the Supporting Information.

### *2.2* *In vitro* experiments

#### 2.2.1 Bacterial adhesion assay

The culture protocols for *Porphyromonas gingivalis*, *Fusobacterium nucleatum* and *Staphylococcus aureus* are detailed in the Supporting Information. The three bacterial strains in the logarithmic growth phase were selected and cocultured with the prepared *Akk*-HA for 24 h, with uncoated HA as the control. Bacteria adhering to the surface of *Akk*-HA or HA were observed by SEM and SYTO 9/PI staining. The amounts of adherent bacteria were quantified via qPCR. The sequences of the primers used for bacterial quantification by qPCR are provided in Table S1 (Supporting Information).

#### 2.2.2 Quantitative polymerase chain reaction (qPCR)

The detailed cell culture protocols can be found in the Supporting Information. RAW264.7 cells were seeded in 6-well plates at a density of 4 × 10^5^ cells per well. Cells were then treated for 24 h with the following: *Akk*-HA and equal amounts of HA, live *A. muciniphila* (L-*Akk*), pasteurized *A. muciniphila* (P-*Akk*), live *Akk*-HA (L-*Akk*-HA), or pasteurized *Akk*-HA (P-*Akk*-HA). In the inflammatory induction model, 2 μg/mL *P. gingivalis*-derived LPS (Sigma-Aldrich, St. Louis, MO, USA) was first added for stimulation for 6 h, followed by treatment in the aforementioned groups for an additional 24 h. Cells treated with only cell culture medium were used as the control. In the osteogenic differentiation experiment, PDLCs and bone marrow-derived mesenchymal stem cells (BMSCs) were seeded in 6-well plates (2.5 × 10^5^ cells per well) overnight and incubated with osteogenic conditioned medium (OCM) prepared differently for 7 days. The OCM preparation process is shown in Fig. 5A. Total RNA was extracted from the cells with a total RNA isolation kit, after which the total RNA was reverse transcribed with HiScript® III-RT SuperMix (Vazyme, Nanjing, China) according to the manufacturer's instructions. The transcript levels of inflammation-related genes, regeneration-related genes and osteogenesis-related genes were evaluated by qPCR. The results were calculated using the 2^−ΔΔCT^ method and normalized to GAPDH. The relevant qPCR primers are shown in Tab. S2, S3 and S4 (Supporting Information).

#### 2.2.3 Cell immunofluorescence

RAW264.7 cells were seeded in 10 mm confocal culture dishes and cultured under inflammatory conditions according to the protocol described in Section 2.2.2. After incubation, cells were fixed with 4 % paraformaldehyde, blocked with 5 % bovine serum albumin (BSA), and then incubated with primary antibodies against CD206 (1:500; Abcam, Cambridge, UK) and CD86 (1:200; Proteintech, Wuhan, China) overnight at 4 °C. The next day, the cells were washed and labelled with the CoraLite594-conjugated Goat Anti-Rabbit IgG (H + L) and Fluorescein (FITC) -conjugated Goat Anti-Rabbit IgG(H + L) (1:100; Proteintech, Wuhan, China) secondary antibodies. Then, the nuclei were stained with DAPI, and the samples were observed by confocal microscopy.

#### 2.2.4 Enzyme-linked immunosorbent assay (ELISA)

RAW264.7 cells were treated as Section 2.2.2. For the mechanistic study, cells were treated together with 25 μM PI3K-IN-1 (MCE, New Jersey, USA). Subsequently, the cell culture supernatants from the different groups were collected, and the secretion levels of IL-10, OSM, transforming growth factor (TGF)-β, and bone morphogenetic protein (BMP)-2 were detected using the respective assay kits (JonLab, Shanghai, China) according to the manufacturer's protocol.

#### 2.2.5 Macrophage-conditioned medium (CM) experiments

RAW264.7 cells were treated as Section 2.2.2. The macrophage-conditioned medium (CM) was collected and centrifuged for 1 h at 300 g to remove residual material and cellular debris. The different CM samples were then mixed with osteogenic medium (low-glucose DMEM supplemented with 10 % foetal bovine serum, 100 IU/mL penicillin‒streptomycin, 100 mM dexamethasone, 10 mM β-phosphoglycerol and 0.05 mM ascorbic acid-2-phosphate) at a 1:2 ratio to obtain the final OCM. PDLCs or BMSCs were cultured with OCM from different groups for 7 and 21 days for subsequent experiments.

#### 2.2.6 Alkaline phosphatase (ALP) and alizarin red S (ARS) staining

PDLCs and BMSCs were seeded in 12-well plates at a density of 1 × 10^5^ cells per well, cultured overnight, and then treated with OCM from different groups. The OCM was refreshed every 3 days. On day 7, the cells were fixed and stained with an ALP assay kit (Beyotime, Shanghai, China). For ARS staining*,* on day 21, fixed cells were stained with 2 % ARS solution for 10 min (Cyagen, Suzhou, China). The cells were subsequently rinsed to remove excess dye and terminate the staining process. Subsequent observations were conducted and the images were captured.

#### 2.2.7 IL-10/OSM neutralization experiment

The conditioned medium from macrophages treated with *Akk*-HA was combined with 2 μg/mL IL-10-neutralizing antibody (MCE, New Jersey, USA) or 2 μg/mL OSM-neutralizing antibody (R&D, Hong Kong, China). PDLCs were cultured with neutralizing OCM and *Akk*-HA-OCM for 7 and 21 days for subsequent experiments.

#### 2.2.8 RNA sequencing assay

RAW264.7 cells were treated as Section 2.2.2. Total RNA was extracted using TRIzol reagent (Thermo Fisher Scientific, CA, USA) according to the manufacturer's instructions. The total RNA quantity and purity were analysed by a Bioanalyzer 2100 and RNA 6000 Nano LabChip Kit (Agilent, CA, USA), and high-quality RNA samples with a RIN >7.0 were used to construct a sequencing library. Sequencing was performed by Shanghai Baiqu Biomedical Technology Co., Ltd., followed by Kyoto Genome Encyclopedia (KEGG) pathway analysis and Gene Ontology (GO) analysis. An absolute value of log2 (fold change) > 1.2 and a FDR value < 0.1 were adopted as the standards for determining significance. The results were visualized using the SRplot online tool [20].

#### 2.2.9 Western blot analysis

RAW264.7 or PDLCs were treated as Section 2.2.2, then the cells were lysed on ice with RIPA lysis buffer supplemented with protease and phosphatase inhibitors. The total protein was subsequently mixed with 5 × SDS sample loading buffer and boiled before electrophoresis. Equal amounts of total protein loading sample were added to each well. After the proteins were separated by SDS-polyacrylamide gel electrophoresis (12 % polyacrylamide gels), they were transferred to polyvinylidene difluoride membranes (Bio-Rad, CA, USA) at a constant current of 140 V for 50 min. The membranes containing proteins were then blocked with 5 % BSA-TBST and incubated with primary antibodies. The primary antibodies used included antibodies against Runt-related transcription factor 2 (Runx2) (1:500, #12556, CST, MA, USA), PI3K (1:1000, #4257, CST, MA, USA), p-PI3K (1:1000, #4228, CST,MA, USA), AKT (1:1000, #4691, CST, MA, USA), and p-AKT (1:2000, #4060, CST, MA, USA). After the membranes were incubated with Goat Anti-Mouse IgM-HRP and Goat Anti-Rabbit IgG (H + L) HRP secondary antibodies (1:50000, Biogot, Nanijng, China), proteins were visualized using a Tanon-5200 chemiluminescence imaging system (Tanon, Shanghai, China).

### *2.3* *In vivo* experiments

#### 2.3.1 Animal experiment procedure

Six-week-old male C57BL/6 mice were obtained from Weitong Lihua Experimental Animal Technology Co., Ltd. (Beijing, China) and maintained on a 12-h light/dark cycle under specific pathogen-free conditions. All animal experiments were authorized by the Animal Ethics Committee and were conducted in accordance with the National Institutes of Health Guide for the Care and Use of Laboratory Animals. Mice were randomly numbered and divided into four groups (n = 12/group): (1) the blank group; (2) the CON group (PBS); (3) the *Akk*-HA group; and (4) the HA group. After periodontitis-induced bone defects were established via silk ligature and localized bacterial application for two weeks, palatal flap surgery was performed at the maxillary second molar for material implantation. One week after surgery, the mice were euthanized, and the maxillary tissue were harvested to assess the antibacterial and immunomodulatory effects of *Akk*-HA (n = 5/group). Four weeks after surgery, the maxillary tissue, and major organs were collected to assess the *in vivo* osteogenic effect and biocompatibility of *Akk*-HA (n = 7/group). The detailed procedures are provided in the Supporting Information.

#### 2.3.2 Bacterial culture experiment

At 7 days after surgery, the palatal tissue of the second molar was detached and immersed in 400 μL of PBS. A bacterial suspension was obtained by vigorous ultrasonic agitation. Five microlitres of the bacterial solution was diluted, spread evenly on an agar plate, and cultivated anaerobically for 48 h. The colonies on the plate were subsequently photographed and counted using ImageJ software (National Institutes of Health, Bethesda, USA).

#### 2.3.3 RNA isolation and qPCR

Gingival tissues were homogenized, and total RNA was extracted using TRIzol (Invitrogen, CA, USA) and reversed transcribed with HiScript® III-RT SuperMix (Vazyme, Nanjing, China). The transcript levels of inflammatory genes were measured by qPCR. qPCR results were calculated via the 2^−ΔΔCT^ method, and the GAPDH gene was used as the control. The relevant qPCR primers are shown in Table S2 (Supporting Information).

#### 2.3.4 Micro-CT analysis

At 4 weeks after surgery, the mice were euthanized, and the maxillae were collected. Maxillae were scanned using a micro-CT imaging system (Bruker Micro-CT, Kontich, Belgium). CTvox and CTan software were used for three-dimensional (3D) reconstruction and imaging analysis, respectively. The extent of alveolar bone loss was evaluated by measuring the distance from the palatal cementoenamel junction to the alveolar bone crest. For the analysis of bone-related parameters, the palatal region of the second molar was designated the region of interest (ROI). The parameters within the ROI, including bone volume fraction (BV/TV), bone mineral density (BMD), and trabecular number (Tb.N), were assessed.

#### 2.3.5 Histological examination

After decalcification with 10 % EDTA, the maxillary tissue was dehydrated, paraffin-embedded, and cut into 4-μm-thick sections. H&E and Masson's trichrome (G1006, Servicebio, Wuhan, China) staining were subsequently conducted according to the manufacturers' instructions. For immunohistochemistry and immunofluorescence analysis, the detailed experimental protocols are provided in the Supporting Information. Histological alterations in the liver, kidney, and spleen were evaluated by H&E staining.

### 2.4 Statistical analysis

Data analysis and visualization were performed using GraphPad Prism 9.0 (GraphPad software, CA, USA). For multiple-group comparisons, one-way analysis of variance (ANOVA) was performed after verifying the normality of the data distribution using the Shapiro-Wilk test (P > 0.05) and the homogeneity of variance using Levene's test (P > 0.05). When the experimental groups were compared with the control group, two-tailed Student's t tests were employed, and the prerequisites of normality and variance homogeneity were confirmed beforehand. If normality assumptions were violated, nonparametric tests (Mann-Whitney U or Kruskal-Wallis) were applied. The results are presented as the mean ± standard deviation (SD). Statistical significance is denoted at three levels: *P < 0.05, **P < 0.01, and ***P < 0.001.

## 3 Results and discussion

### 3.1 Preparation and characterization of *Akk*-HA

**Fig. 1:**
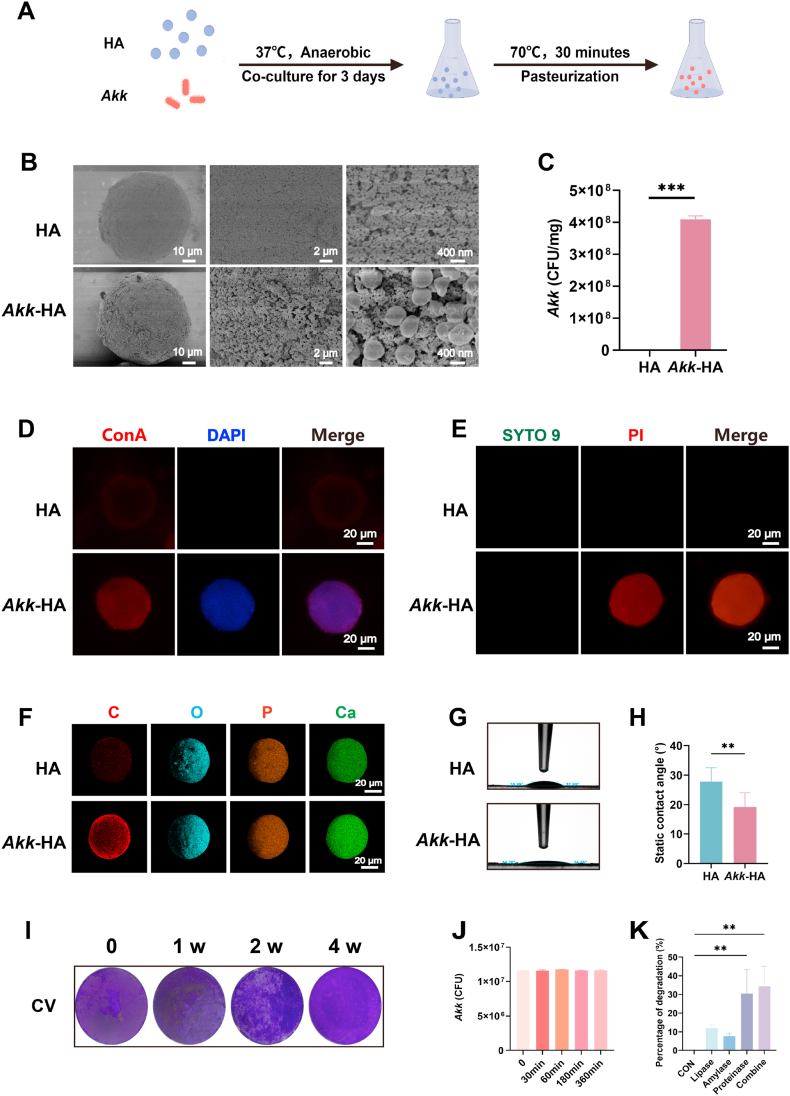
Preparation and characterization of *Akk*-HA. (A) Flowchart of *Akk*-HA preparation. (B) SEM images, (C) qPCR quantitative results (n = 3), (D) Con A/DAPI staining and (E) SYTO 9/PI staining images of HA and *Akk*-HA. (F) EDS elemental mapping. (G) Static contact angle measurement. (H) Static contact angle quantification (n = 4). (I) Crystal violet staining images of *A. muciniphila* biofilms. (J) Stability evaluation of *Akk*-HA in dynamic scouring conditions (n = 3). (K) Effects of enzymes (proteinase, amylase, lipase, and three enzymes in combination) on the biofilm (n = 3). **P < 0.01, and ***P < 0.001.

*A.muciniphila* were cocultured with HA for 3 days and subsequently inactivated through pasteurization (70 °C, 30 min; Fig. 1A). SEM revealed that elliptical *A. muciniphila* were attached to the HA surface as single or paired bacteria (Fig. 1B). qPCR analysis revealed that the amount of *A. muciniphila* coating the HA was approximately 4.1 × 10^8^ CFU per mg HA (Fig. 1C). Concanavalin A (Con A), a lectin specific for biofilm glycoproteins, combined with DAPI staining demonstrated the formation of a dense *A. muciniphila* biofilm on HA (Fig. 1D). SYTO 9/PI staining of *Akk*-HA revealed predominantly red fluorescence, confirming successful inactivation by pasteurization (Fig. 1E). In addition, through energy-dispersive X-ray spectroscopy (EDS) elemental analysis, we observed that the carbon content in *Akk*-HA was significantly greater than that in uncoated HA, confirming the stable colonization of *A. muciniphila* on the HA surface (Fig. 1F). Mechanical characterization of *Akk*-HA revealed that the average maximum loading of the *A. muciniphila* biofilm reached 199.86 ± 0.07 μN, the average Young's modulus was 24.81 ± 22.36 GPa, and the nanoindentation hardness was 1.39 ± 0.74 GPa. Moreover, *A. muciniphila* decreased the contact angle of HA, indicating a significant increase in surface hydrophilicity (Fig. 1G–H). To evaluate the long-term stability of *Akk*-HA, the formed *A. muciniphila* biofilm was immersed in PBS and stained with crystal violet (CV) at different time points. As shown in Fig. 1I, the *A. muciniphila* biofilm still maintained its intact structure after 4 weeks of soaking. Moreover, to evaluate the stability of the material in the physiological environment, we designed a dynamic scouring experiment. At a flow rate of 1 mL/min, the *Akk*-HA sample maintained its initial loading in the PBS solution for 6 h (Fig. 1J). Finally, to assess the degradation rate of the *A. muciniphila* biofilm, the biofilm was treated with proteinase, amylase, lipase, and a combination of all the enzymes. The results demonstrated that proteinase significantly compromised biofilm integrity, reducing it by 30.9 %, whereas lipase and amylase had only minor effects, with reductions of 11.9 % and 7.7 %, respectively; these findings strongly suggest that glycoproteins play a predominant role in maintaining the structural stability of *A. muciniphila* biofilms (Fig. 1K). Based on this finding, *Akk*-HA may leverage the highly expressed protease microenvironment in periodontitis lesions to establish an enzymatically trigger-degradable biofilm system.

Pasteurization is a traditional heat treatment process applied to food; probiotics processed by it can still maintain their specific activity. Studies have demonstrated that inactivated *Lactobacillus reuteri* can regulate the balance of the intestinal microbiota, thereby alleviating inflammation and enhancing host immunity [21]. Plovier H. et al. reported that the surface proteins of *A. muciniphila* can retain their original structural properties after pasteurization and can exert immunomodulatory and metabolic effects [18]. In bone replacement materials, the use of pasteurized *A*.*muciniphila*-which possesses inherent safety and niche specificity-effectively avoids potential risks associated with viable bacterial applications, such as bacteremia-induced systemic infections or excessive immunostimulatory responses [22], and facilitates standardized production and storage. Compared with alternative HA modification strategies, pasteurized probiotic-based surface functionalization demonstrated superior biocompatibility by significantly reducing the concentration-dependent cytotoxicity and metabolic disturbances associated with conventional antibiotics or metal ion-modified counterparts [23]. Critically, it also effectively avoids the inherent limitations of probiotics inactivated by ultraviolet, which may induce cross-linking or denaturation of bacterial surface proteins through DNA/RNA damage, thereby reducing antigenicity and damaging immune recognition [24].

### *3.2* *Akk*-HA resisted the adhesion of pathogens

**Fig. 2:**
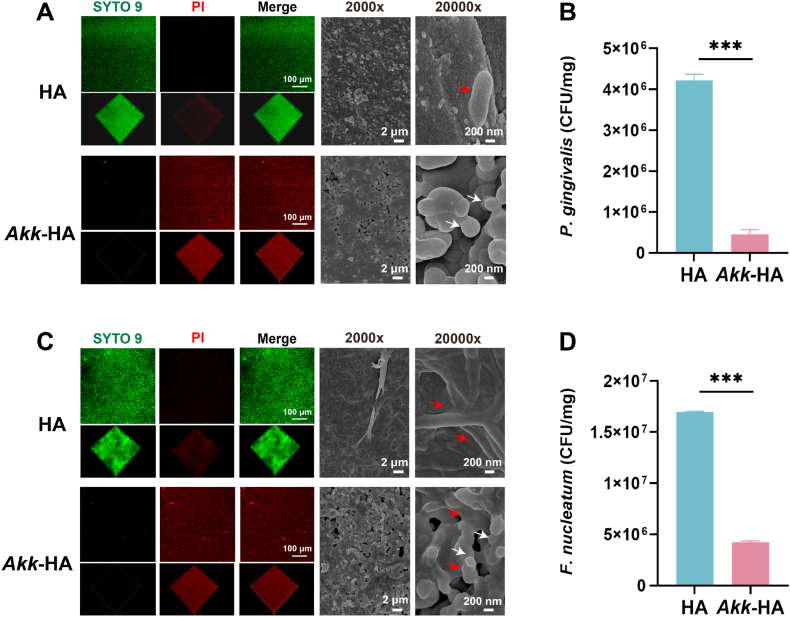
Anti-adhesion performance of *Akk*-HA against pathogenic bacteria. SYTO 9/PI staining images, typical SEM images and quantitative qPCR results for *P. gingivalis* (A–B) and *F. nucleatum* (C–D) (n = 3). Pathogenic bacteria are marked with red arrows and *A. muciniphila* with white arrows. ***P < 0.001.

The anti-adhesion properties of *Akk*-HA were subsequently evaluated. *P. gingivalis* and *F. nucleatum* are recognized as common pathogens associated with periodontitis and peri-implantitis [25,26]. *Akk*-HA and HA were cocultured with *P. gingivalis* and *F. nucleatum* for 24 h. In SYTO 9/PI staining, red fluorescence represents inactivated *A. muciniphila*, while green fluorescence indicates *P. gingivalis* or *F. nucleatum*. The results revealed that HA displayed extensive green fluorescence, indicating high susceptibility to adhesion by *P. gingivalis* and *F. nucleatum*. Conversely, *Akk*-HA exhibited pronounced red fluorescence with minimal green fluorescence, demonstrating substantially reduced adhesion of *P. gingivalis* and *F. nucleatum*. Representative SEM images similarly revealed numerous *P. gingivalis* and *F. nucleatum* adhering to the surface of HA, whereas these bacteria were almost absent on the surface of *Akk*-HA (Fig. 2A–C). The results of qPCR further confirmed the above observations. Compared with that on HA, the degree of *P. gingivalis* colonization on the *Akk*-HA surface was 89.34 % lower (Fig. 2B). *Akk*-HA exhibited similar antiadhesion effects against *F. nucleatum*, with a 75.18 % reduction of colonization on its surface (Fig. 2D). In addition to periodontal pathogens, we assessed antiadhesion activity against *S. aureus* because of its clinical relevance in infected bone defects (Fig. S1, Supporting Information) [27]. The results showed that *Akk*-HA exhibited a significant antiadhesion effect against to *S. aureus*. However, the current findings remain limited to three specific pathogens; confirming truly broad-spectrum anti-adhesive properties requires systematic validation with a wider range of microorganisms, including fungi and other Gram-positive/negative bacteria.

Studies have demonstrated that probiotic bacteria such as *Lactobacillus rhamnosus GG* and *Lactiplantibacillus plantarum ZJ316* can produce biosurfactants or metabolites to prevent the adhesion of pathogens [28,29]. Tan L et al. reported that titanium implants coated with a UV-inactivated *Lactobacillus casei* biofilm similarly confer resistance to the colonization and adhesion of methicillin-resistant *S. aureus* [30]. Liu et al. reported that *A. muciniphila* could inhibit pathogen growth, bacterial biofilm formation, and bacterial adherence to and invasion of intestinal epithelial cells. Various organic acids (specifically acetate and propionate) produced by *A. muciniphila* might be responsible for its antibiofilm and antivirulence properties [31]. Moreover, in addition to those organic acids, certain exopolysaccharides (EPSs) produced by *A. muciniphila* may also mediate antiadhesion effects. EPSs from the probiotic *Lactobacillus paracasei* have been shown to prevent the adhesion of *E. coli* to Caco-2 cells [32]. Future studies should focus on elucidating the anti-adhesion mechanisms of *Akk*-HA.

### *3.3* *Akk*-HA regulated the expression of pro-inflammatory and regenerative factors in macrophages

**Fig. 3:**
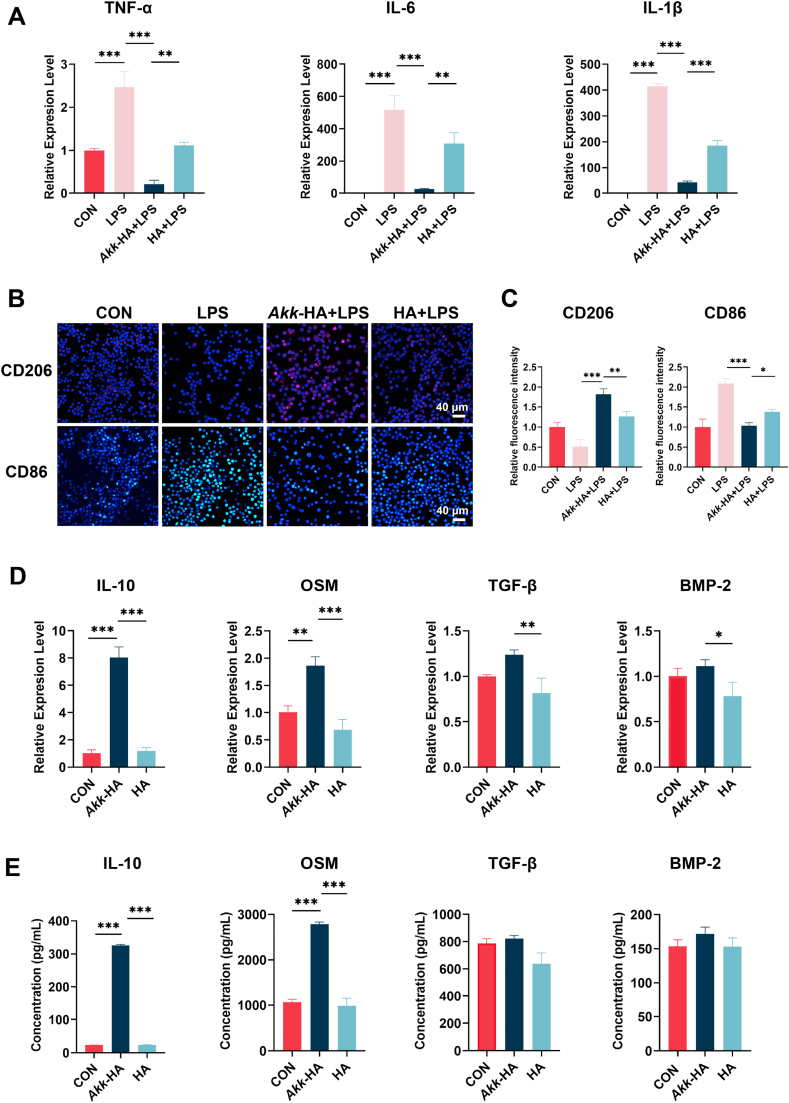
Regulatory effects of *Akk*-HA on macrophages. (A) Relative mRNA expression levels of inflammatory cytokines under inflammatory conditions (n = 3). (B) Immunofluorescence staining for macrophage phenotypic markers. (C) Quantitative analysis of relative fluorescence intensity based on fluorescence images (n = 3). (D) Relative mRNA expression levels and (E) protein expression levels of regenerative cytokines in the culture supernatant (n = 3). *P < 0.05, **P < 0.01, and ***P < 0.001.

The host immune response is a critical determinant of the ultimate fate of implanted biomaterials. In the treatment of periodontal defects, the first cells to interact with the implanted material are immune cells derived from the bloodstream. This characteristic makes the regulation of the immune microenvironment a central consideration in biomaterial design. As central regulators of the immune response, macrophages play a pivotal role in the healing of infected wounds [33,34]. We initially assessed the effects of varying concentrations of *Akk*-HA on the viability of macrophages. The results indicated that within the observed concentration range, *Akk*-HA demonstrated no significant cytotoxic effects (Fig. S2, Supporting Information). In preliminary pilot experiments, we observed that a concentration of 500 ng/mL elicited an attenuated inflammatory response but better osteogenic effects (Fig. S3, Supporting Information). Based on these findings, this concentration was selected for further experimental validation. Under LPS-simulated inflammatory conditions, inactivated *Akk*-HA significantly downregulated the expression of inflammatory cytokines (TNF-α, IL-6 and IL-1β) (Fig. 3A). Additionally, *Akk*-HA increased the expression of CD206 (an anti-inflammatory marker of alternative activation) while reducing the expression of CD86 (an inflammatory marker of classical activation) (Fig. 3B–C). Previous studies have shown that *A. muciniphila* can alleviate intestinal inflammation in mice administered DSS by decreasing the expression levels of inflammatory factors [35]. Similarly, Huck O et al. demonstrated that *A. muciniphila* could alleviate periodontal inflammation resulting from *P. gingivalis* infection [13]. The significant anti-inflammatory effects of *A. muciniphila* under inflammatory conditions were further confirmed in this study.

Numerous studies have demonstrated that macrophages can stimulate the osteogenic differentiation of BMSCs by secreting cytokines, including IL-10, OSM, TGF-β, and BMP-2, thereby upregulating relevant signalling pathways [[36], [37], [38]]. We next investigated the expression levels of these tissue repair signals. The results of qPCR revealed that *Akk*-HA significantly increased the levels of IL-10 and OSM, while the expression of TGF-β and BMP-2 did not significantly change (Fig. 3D). The levels of these cytokines in macrophage culture supernatants from different groups, as determined by ELISA, were consistent with the qPCR results (Fig. 3E). Although the advantages of inactivated *A. muciniphila* in regulating inflammation and metabolism have been reported [39,40], whether pasteurized *A. muciniphila* is advantageous for promoting bone regeneration remains unclear. Thus, the effects of HA alone, live or pasteurized *A. muciniphila*, and live or pasteurized *Akk*-HA were compared. The results indicated that compared with the live counterpart, pasteurized *A. muciniphila* exhibited enhanced anti-inflammatory effects and increased expression of regenerative factors. When the surface of HA was modified with *A. muciniphila*, these advantages were even more pronounced (Fig. S4, Supporting Information).

Previous studies have demonstrated that IL-10 or OSM may play an instrumental role in regulating bone regeneration. The bone-like nano-HA particles fabricated by Mahon et al. facilitated osteogenic differentiation and mineral deposition in BMSCs by enhancing the secretion of IL-10 from macrophages [41]. Wang et al. demonstrated that Mg-activated macrophages can regulate osteogenic activity through the OSM/gp130 signalling pathway [42]. As a bone replacement material, *Akk*-HA might be capable of inducing macrophages to transition towards a regenerative phenotype with high levels of IL-10 and OSM.

### 3.4 Macrophage supernatant from *Akk*-HA cultures promoted osteogenic differentiation

**Fig. 4:**
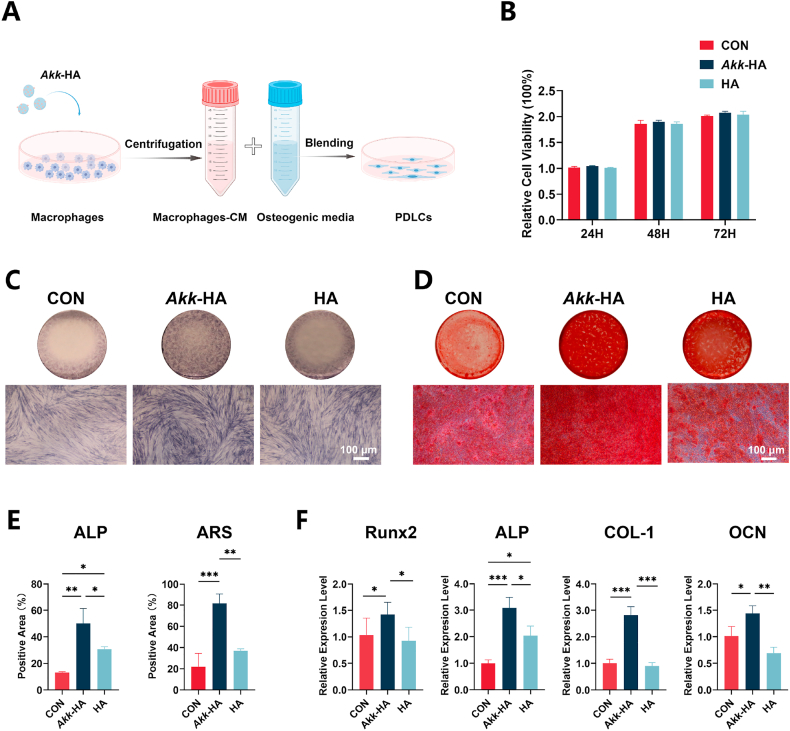
*Akk*-HA-treated macrophage culture supernatant promoted the osteogenic differentiation of PDLCs. (A) Flowchart for the use of different macrophage-CM samples blended with osteogenic media for the osteogenic induction of PDLCs. (B) The relative cell viability of PDLCs in different OCM samples (n = 3). (C) Representative images of ALP staining after 7 days in PDLCs cultured under different OCM samples. (D) Representative images of ARS staining after 21 days in PDLCs cultured under different OCM samples. (E) Semi-quantitative results of ALP and ARS. (F) Relative mRNA expression levels of osteogenic genes in PDLCs cultured with different OCM samples (n = 3). *P < 0.05, **P < 0.01, and ***P < 0.001.

PDLCs exhibit biological characteristics similar to those of BMSCs and play a critical role in alveolar bone repair [43,44]. The effects of macrophage supernatant from *Akk*-HA cultures on PDLCs were evaluated (Fig. 4A). The CCK-8 results revealed that neither *Akk*-HA-OCM nor HA-OCM had cytotoxic effects on PDLCs in terms of viability (Fig. 4B). ALP serves as a crucial marker for the early stage of osteogenic differentiation [45], and the results demonstrated the highest ALP activity in the *Akk*-HA group. Additionally, the effect of *Akk*-HA-OCM on extracellular matrix mineral deposition was examined by ARS staining. The results revealed that a considerable number of mineralized nodules in the matrix of the *Akk*-HA group were fused into sheets, while there were fewer nodules in the HA group, which were scattered (Fig. 4C). qPCR analysis of relevant osteogenic genes revealed that compared with the other groups, the *Akk*-HA group presented significantly higher levels of Runx2, ALP, collagen I (COL-1), and osteocalcin (OCN) (Fig. 4D). It is worth noting that although this study focuses on the immunomodulatory mechanism of *Akk*-HA, its potential direct effects on PDLCs should not be overlooked. This dual mechanism, which combines indirect immunomodulation with direct cellular stimulation, warrants in-depth investigation in future studies. To further systematically evaluate the osteoimmunomodulatory properties of *Akk*-HA, we assessed its effects on the osteogenic differentiation of murine BMSCs. The results revealed that *Akk*-HA upregulated osteogenic gene expression and enhanced mineralized nodule formation in BMSCs (Fig. S5, Supporting Information). These results demonstrate that the osteoimmunomodulatory properties of *Akk*-HA effectively enhance the osteogenic differentiation of both dental tissue-derived and bone marrow-derived stem cells.

### 3.5 Macrophage-derived IL-10 mediates the osteogenic effect of *Akk*-HA on PDLCs

**Fig. 5:**
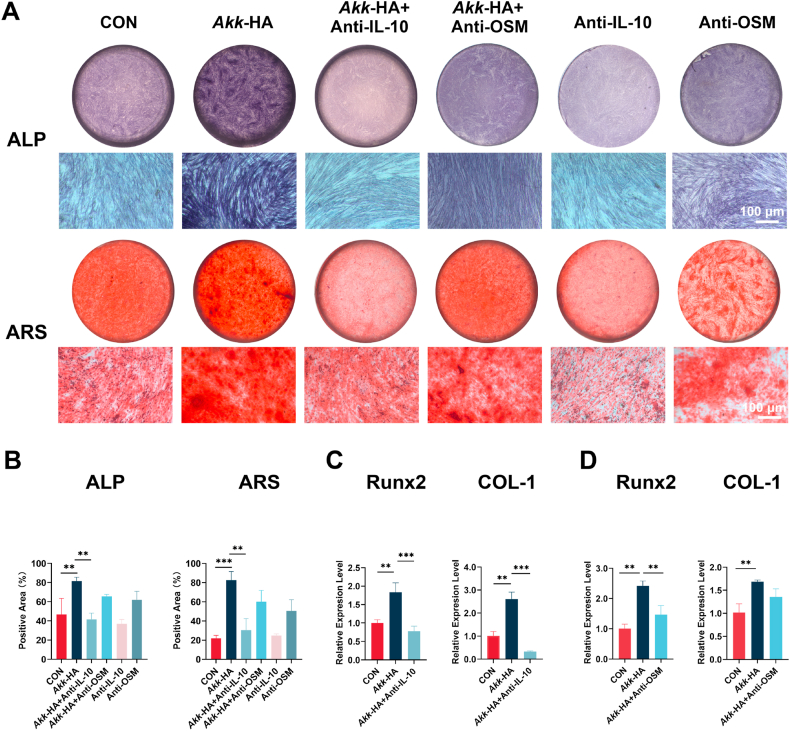
*Akk*-HA-OCM promoted osteogenic differentiation and mineralization in PDLCs in an IL-10-dependent manner. (A) Representative images of ALP staining after 7 days and ARS staining after 21 days in PDLCs cultured in OCM subjected to different neutralization strategies. (B) Semi-quantitative results of ALP and ARS staining. (C–D) Relative mRNA expression levels of osteogenic genes after the neutralization of IL-10 or OSM (n = 3). **P < 0.01, and ***P < 0.001.

To identify the key cytokines through which *Akk*-HA promoted the osteogenic differentiation of PDLCs, cytokine neutralization experiments were conducted. When the expression of IL-10 in *Akk*-HA-OCM was neutralized, ALP activity and ARS staining were significantly weaker, and Runx2 and COL-1 gene expression was reduced considerably. Conversely, while OSM neutralization downregulated the expression of osteogenesis-related genes (e.g., Runx2), its inhibitory effects on COL-1 expression, ALP activity, and mineralized nodule formation were markedly weaker than those of IL-10 neutralization (Fig. 5). These results indicate that the pro-osteogenic effect of the supernatant from *Akk*-HA-stimulated macrophages on PDLCs was significantly attenuated upon IL-10 neutralization, suggesting that IL-10 is a key mediator in this process. IL-10, a cytokine with dual functions in immune regulation and tissue repair, plays a crucial role in regulating the homeostasis of the bone tissue microenvironment [46]. Bone repair and regeneration can be facilitated by specifically enhancing the production of IL-10, guiding the anti-inflammatory innate immune response associated with tissue repair and regeneration [41]. The classical STAT3 signalling pathway may be the primary mechanism through which IL-10 exerts its regulatory effect on bone metabolism [47]. Chen et al. also reported that IL-10 can significantly increase the osteogenic differentiation potential of mesenchymal stem cells by specifically activating the p38/MAPK signalling pathway [48]. Consistent with these investigations, our study demonstrates that IL-10 plays important role in the osteogenic differentiation of PDLCs.

### *3.6* *Akk*-HA promoted IL-10 production by activating the PI3K/AKT signalling pathway

**Fig. 6:**
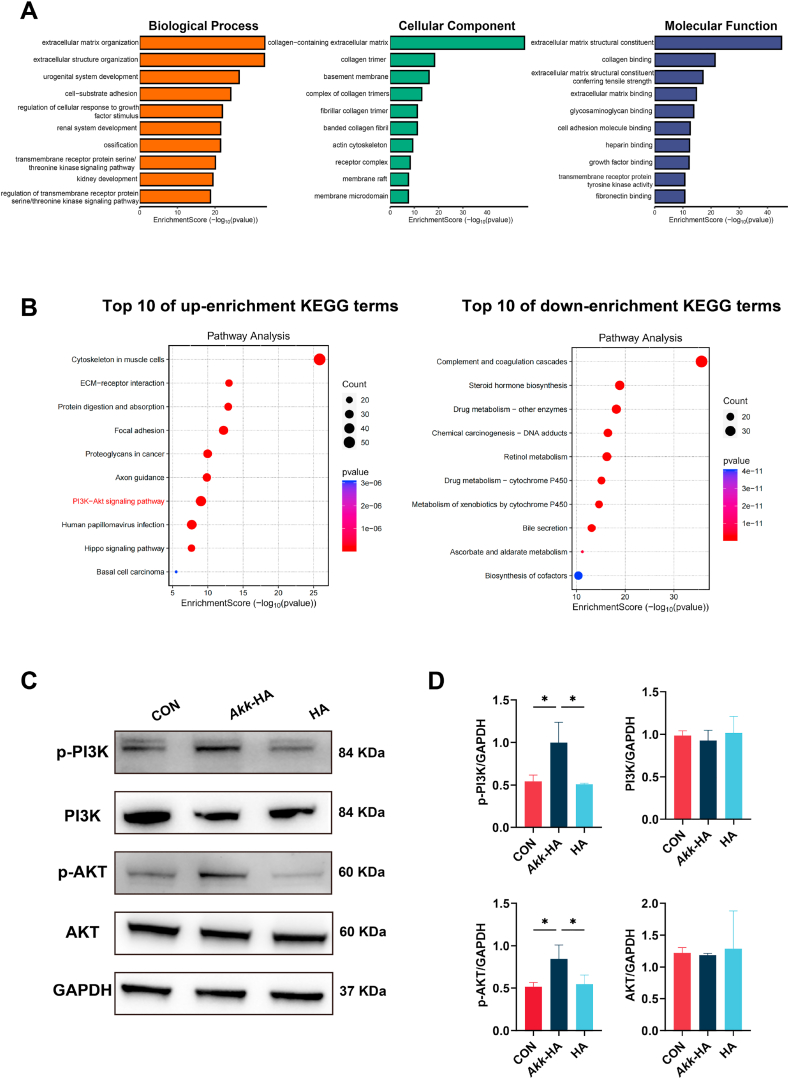
*Akk*-HA promoted IL-10 production by activating the PI3K/AKT signalling pathway. (A) GO analysis includes functional pathways, biological processes, and cellular components. (B) KEGG enrichment analysis. (C) Representative Western blot images of PI3K/AKT signalling pathway activity. (D) Relative quantification of PI3K/AKT signalling pathway protein expression (n = 3). *P < 0.05.

To further investigate the potential mechanism through which *Akk*-HA promotes IL-10 production, RNA sequencing analysis was performed. RNA-seq analysis, visualized by a volcano plot and a circular clustering heatmap of differentially expressed genes (DEGs), revealed widespread transcriptomic changes in macrophages treated with *Akk*-HA (Fig. S6, Supporting Information). Furthermore, GO analysis of differentially expressed genes between the two groups revealed that the expression of genes related to wound healing, collagen-containing extracellular matrix, and extracellular matrix structural components significantly differed between the *Akk*-HA group and control group (Fig. 6A). These genes are related to tissue repair or bone formation and mineralization [49,50]. We subsequently performed a KEGG enrichment analysis and found that the phosphatidylinositol-3-kinase/protein kinase B pathway (PI3K/AKT) was significantly upregulated in the *Akk*-HA group (Fig. 6B). The PI3K/AKT pathway is a well-established signalling cascade in cellular biology that is closely associated with phosphatidylinositol and serves as a pivotal pathway for osteoblast differentiation [51]. A prior study demonstrated that activation of the PI3K/AKT signalling pathway stimulates the expression of the anti-inflammatory factor IL-10 in macrophages [52,53]. The Western blot results further supported these findings and revealed that compared with the CON group and HA group, the *Akk*-HA group exhibited significant increases in the expression of p-PI3K and p-AKT (Fig. 6C–D). Thus, we hypothesized that *Akk*-HA might increase IL-10 expression by activating the PI3K/AKT signalling pathway, thereby facilitating the osteogenic differentiation of PDLCs.

**Fig. 7:**
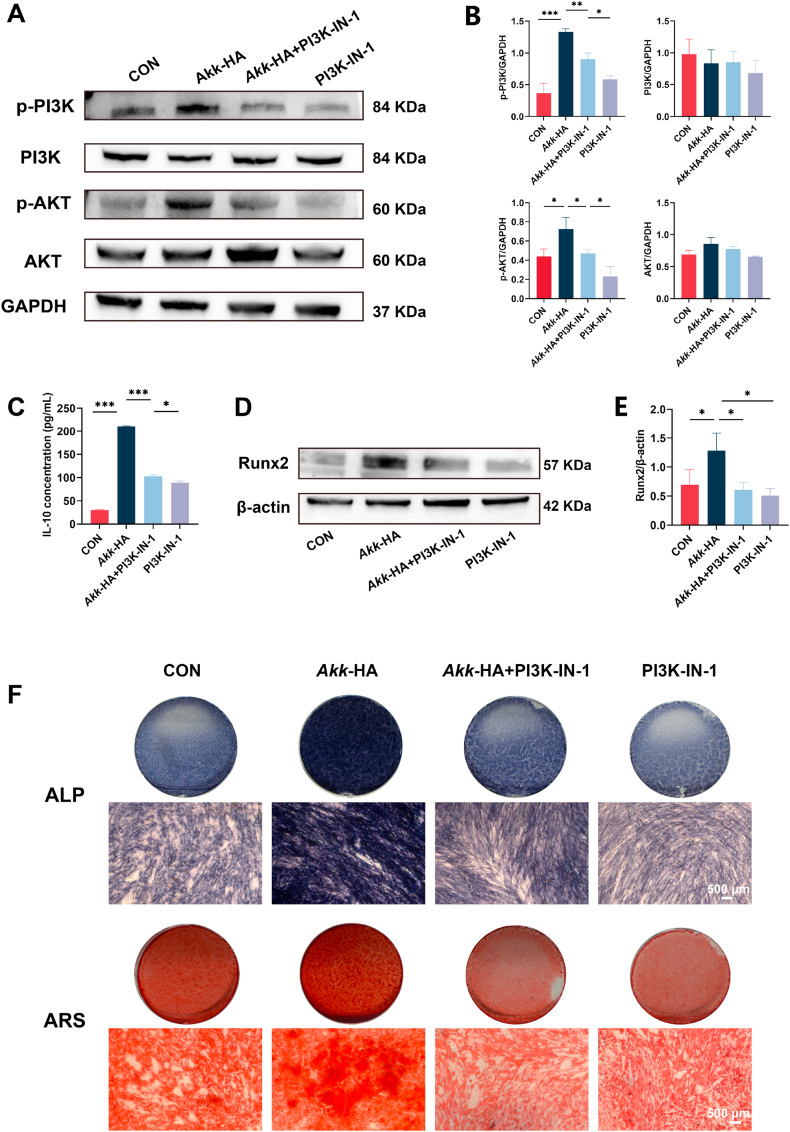
IL-10 downregulation-mediated osteogenesis following PI3K/AKT inhibition. (A–B) Representative Western blot images and relative quantification of PI3K/AKT signalling pathway protein expression (n = 3). (C) Expression level of IL-10 protein in the supernatant of cells treated with PI3K-IN-1 (n = 3). (D–E) Representative Western blot images and relative quantification of Runx2 expression in PDLCs (n = 3). (F) ALP staining of PDLCs at 7 days and ARS staining at 21 days. *P < 0.05, **P < 0.01, and ***P < 0.001.

The key role of the PI3K/AKT signalling pathway was further verified using a PI3K/AKT pathway inhibitor (PI3K-IN-1). The Western blot results revealed that the expression of p-PI3K and p-AKT decreased following treatment with PI3K-IN-1 (Fig. 7A–B). Significantly, the ELISA results revealed a significant decrease in the expression of IL-10 in the macrophage supernatant after inhibition of the PI3K/AKT pathway (Fig. 7C). To further evaluate the impact of PI3K/AKT pathway blockade on the *Akk*-HA-mediated macrophage regulation of osteogenic differentiation, we prepared OCM using the supernatant from macrophage cultures treated with PI3K-IN-1. PDLCs exhibited significantly suppressed osteogenic capacity, and Runx2 expression was downregulated in the *Akk*-HA + PI3K-IN-1-treated PDLCs versus *Akk*-HA-treated PDLCs (Fig. 7D–E). Concordantly, ALP activity was decreased on day 7, whereas on day 21, ARS staining revealed reduced mineralization, with sparse, disorganized nodules after PI3K-IN-1 treatment (Fig. 7F).

The induction of the PI3K/AKT pathway by activating receptors, such as TLRs, has been shown to act as an inhibitory feedback mechanism that limits inflammatory cytokine production [52]. Studies have shown that the AKT-mediated phosphorylation and inactivation of GSK3 releases the IL-10 gene from GSK3-mediated repression, resulting in increased IL-10 production [54,55]. Similarly, a recent study revealed that the aminoacyl-tRNA synthetases (AmTARSs) generated by *A. muciniphila* can activate the MAPK and PI3K/AKT signalling pathways through specific interactions with TLR2, thereby facilitating the production of IL-10 [46]. These findings demonstrate that *Akk*-HA promotes macrophage IL-10 secretion through the activation of the PI3K/AKT signalling pathway and further enhances the osteogenic differentiation of PDLCs.

### *3.7* *Akk*-HA resists pathogen colonization and modulates immune responses *in vivo*

**Fig. 8:**
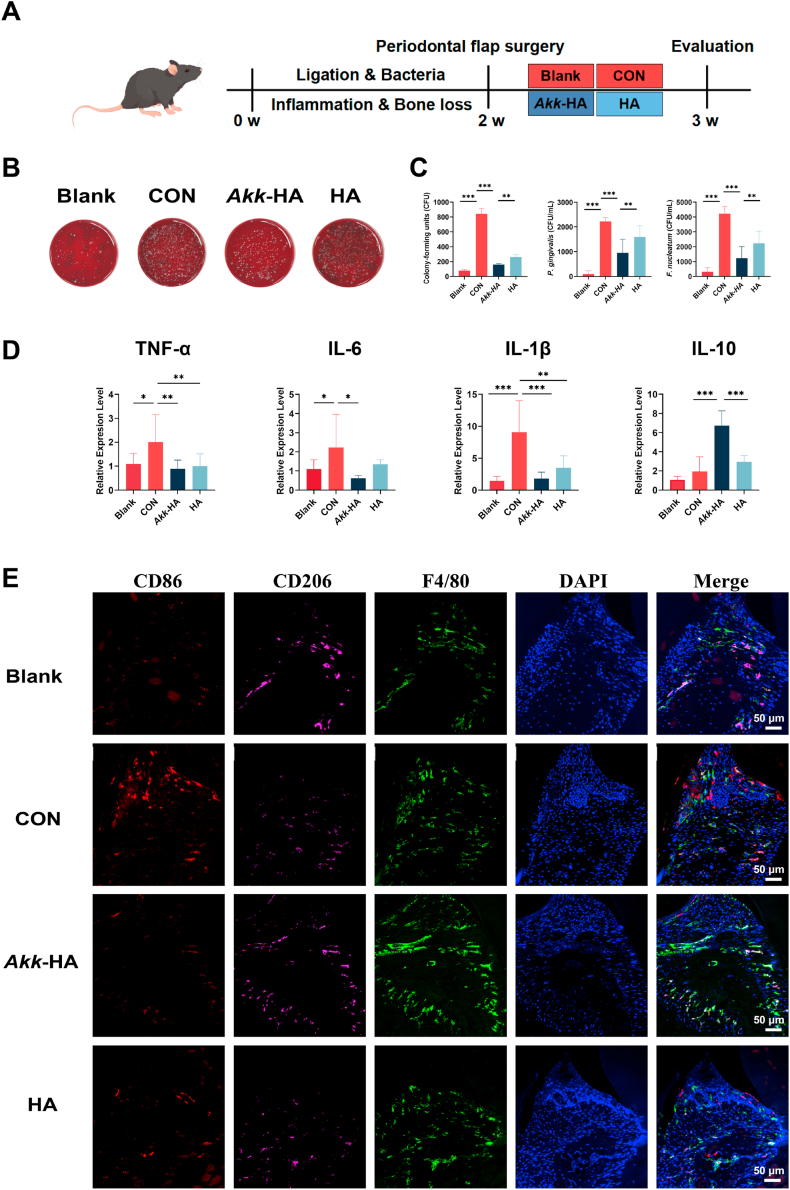
The anti-adhesion effect of *Akk*-HA and its regulatory effect on macrophage during the early healing phase. (A) Flow chart of the animal experiment. (B) Photographs of bacterial colony-forming units obtained from the palatal alveolar bone of second molars treated under various experimental conditions. (C) Bacterial colony counts and quantitative results for *P. gingivalis and F. nucleatum* (n = 4)*.* (D) Relative mRNA expression levels of inflammatory factors in gingival tissues (n = 5). (E) Representative immunofluorescence images showing the expression of macrophage markers in periodontal tissues. *P < 0.05, **P < 0.01, and ***P < 0.001.

To evaluate the therapeutic potential of *Akk*-HA *in vivo*. Infected periodontal bone defects were established via silk ligature combined with the local inoculation of *P. gingivalis* and *F. nucleatum* around the maxillary second molars in a mouse model. The detailed experimental procedure is illustrated in Fig. 8A. At 1 week after flap surgery, the maxillary second molar surgical site exhibited a significantly lower bacterial load in the *Akk*-HA group versus the CON and HA control groups (Fig. 8B). The qPCR results indicated an 11-fold increase in bacterial counts in the CON group compared with the blank group, while the increase was only 2-fold greater in the *Akk*-HA group (Fig. 8C). Furthermore, the analysis of inflammatory factor levels in gingival tissue further confirmed the interventional effect of *Akk*-HA. qPCR results demonstrated that, compared with the CON group, the mRNA expression of pro-inflammatory factors IL-1β, IL-6, and TNF-α was significantly downregulated in the *Akk*-HA group, while the expression of the anti-inflammatory factor IL-10 was markedly upregulated (Fig. 8D). Meanwhile, in the defect microenvironment, the expression of M1 macrophages (CD86^+^F4/80^+^) significantly decreased in the *Akk*-HA group, whereas the expression of M2 macrophages (CD206^+^F4/80^+^) correspondingly increased (Fig. 8E). These findings suggest that *Akk*-HA is capable of resisting colonization by pathogenic bacteria and subsequently ameliorating gingival inflammation in mice.

Resistance to the adhesion of pathogenic bacteria is a prerequisite for the establishment of a favourable osteogenic microenvironment. To prevent bacterial infection, various strategies, including the integration of biomaterials with antibiotics, metal nanoparticles, antimicrobial peptides, and biological extracts, have been used [56]. Although antibiotic-loaded HA materials can effectively inhibit the adhesion and colonization of pathogenic bacteria, their biological properties are significantly compromised by increased antibiotic loading [57]. *Akk*-HA not only inhibits bacterial adhesion but also avoids the risk of antibiotic resistance and potential exacerbation of inflammatory responses. In addition, metal-ion-coated materials such as silver ion-doped nano-HA combine broad-spectrum antimicrobial properties with the ability to promote the osteogenic differentiation of BMSCs [23]. However, the concentration-dependent release of silver ions may cause cytotoxicity [58]. Thus, inactivated probiotic coatings have good biocompatibility and biosafety and can avoid resistance caused by antibiotic use, offering a promising surface modification strategy for implanted biomaterials.

### *3.8* *Akk*-HA promotes the repair of infected periodontal bone defects

**Fig. 9:**
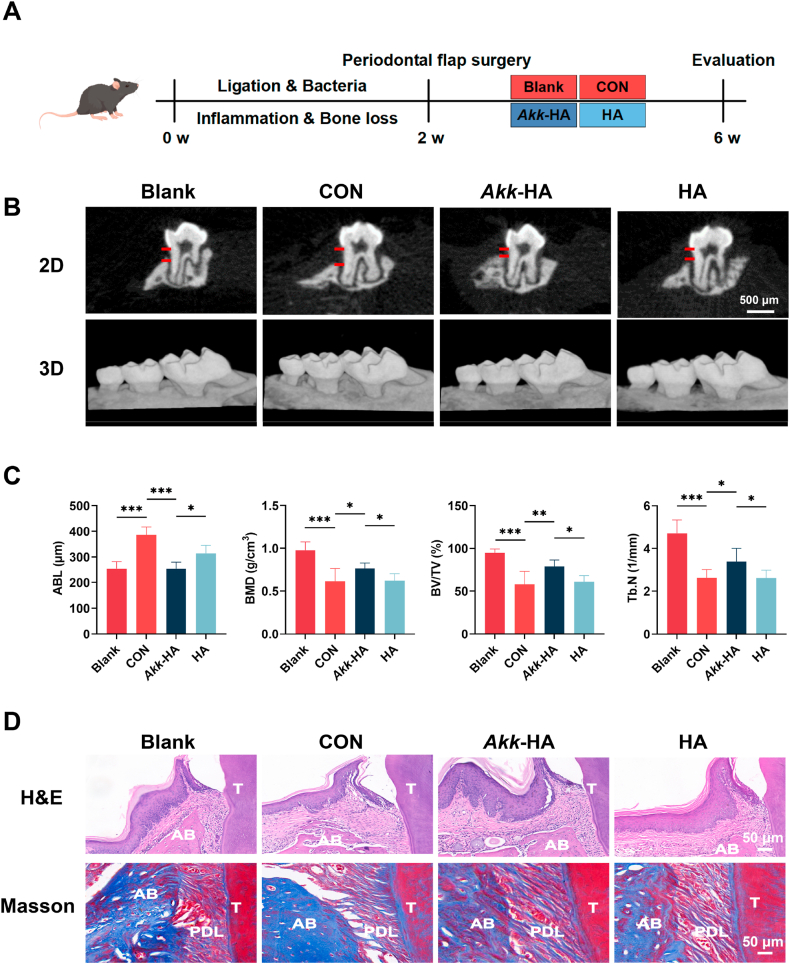
The therapeutic effects of *Akk*-HA on infected periodontal bone defects. (A) Flow chart of the animal experiment. (B) Alveolar bone loss (ABL) at the second molar in the different groups. The red lines indicate the cementoenamel junction and the alveolar bone crest. (C) Quantitative analysis of bone-related parameters (n = 7). (D) H&E and Masson staining of samples from the different groups. AB represents the palatal alveolar bone of the second molar, T represents the second molar, and PDL represents the periodontal ligament. *P < 0.05, **P < 0.01, and ***P < 0.001.

**Fig. 10:**
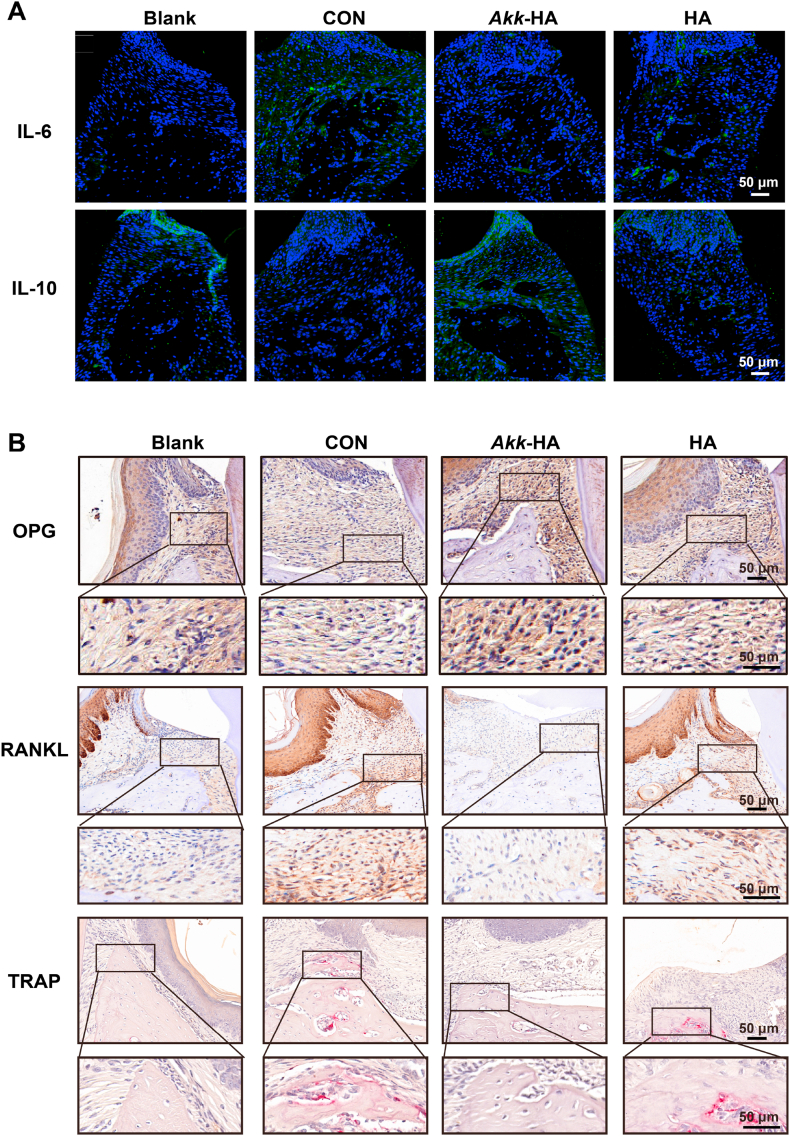
Detection of inflammatory cytokines and bone-related markers in periodontal tissues. (A) Representative images of Immunofluorescence staining of IL-6 and IL-10. (B) Representative images of immunohistochemical staining of OPG and RANKL, and TRAP staining.

The therapeutic effects of *Akk*-HA on periodontal bone defects were observed 4 weeks after periodontal flap surgery (Fig. 9A). Micro-CT revealed increased alveolar bone height and density at the maxillary second molars in the experimental groups versus the control groups (Fig. 9B), with *Akk*-HA showing the lowest degree of alveolar bone loss (ABL) among the groups. Additionally, bone parameters (BV/TV, BMD, and Tb.N) significantly improved, indicating regenerative efficacy (Fig. 9C). Histologically, H&E staining revealed that compared with CON and HA, *Akk*-HA reduced inflammatory cell infiltration and promoted new bone formation. Masson staining revealed noticeable deposition of an orderly collagen matrix in the collagen fibres of the *Akk*-HA group, indicating that the repaired bone tissues were more mature (Fig. 9D). Immunofluorescence analysis demonstrated that *Akk*-HA significantly upregulates IL-10 expression while downregulating IL-6 expression in periodontal tissues (Fig. 10A). Immunohistochemical analysis revealed that *Akk*-HA treatment upregulates the expression of OPG while downregulating RANKL in periodontal tissues. TRAP staining confirmed a reduction in osteoclast numbers in the *Akk*-HA treated group compared to the CON (Fig. 10B). These results reveal that *Akk*-HA shapes a conducive microenvironment for bone regeneration by restoring osteoimmune homeostasis. Moreover, no pathological changes in major organs confirmed biosafety (Fig. S7, Supporting Information). These findings indicate that *Akk*-HA creates an optimal microenvironment for bone repair by facilitating the synergistic interaction of multiple factors.

*A. muciniphila* itself and its active components might play a role in facilitating periodontal bone repair through endogenous regulation of the bone immune microenvironment. It has been demonstrated that *A. muciniphila* promotes fracture healing and H-type angiogenesis by relieving systemic inflammation in mice [59]. Additionally, *A. muciniphila* derived from the gut flora of children has been shown to exert a bone-preserving function by secreting extracellular vesicles, facilitating osteoblast bone formation and restraining osteoclast bone resorption [60]. In this study, *Akk*-HA could prevent pathogen colonization, reduce inflammation, and establishing a favourable osteogenic microenvironment. Furthermore, it might increase the secretion of IL-10 by macrophages and facilitate the osteoblastic differentiation of stem cells.

Although *Akk*-HA has shown promising potential as a biomaterial for treating infected bone defects, numerous challenges still exist in its practical application and clinical translation. During the preclinical validation stage, it is essential to systematically evaluate the long-term efficacy of the material using large animal models (e.g., canine, porcine). This necessitates establishing a multidimensional evaluation system that incorporates periodic sampling to analyse the in v*ivo* status of the material, histological assessment of the bone defect area, and dynamic monitoring of blood biochemical indicators, thereby enabling a comprehensive investigation of the material's long-term stability and chronic immune response characteristics. It should be noted that although no significant off-target inflammatory responses were observed during the experimental period, this conclusion is limited by the study duration, and long-term potential risks require further validation through more sensitive imaging techniques and organ-specific inflammatory factor analyses.

This study has the following limitations. Firstly, at the mechanistic level, the specific cellular details or molecular mechanisms underlying the interaction between *Akk*-HA and macrophages, as well as its antibacterial effects have not been elucidated. Studies have demonstrated that *A. muciniphila* can interact with host cells and regulate host signalling through various bioactive molecules, such as, secretions, metabolites, and extracellular vesicles [61,62]. Future studies need to combine bioassay-guided fractionation with LC‒MS/MS profiling to identify the principal bioactive constituents in *Akk*-HA and engineered *A. muciniphila-*coated HA constructs. Secondly, in this study, the surface modification density was controlled by adjusting the culture duration, but future advancements may require more precise methods, such as microfluidic technology or nanoscale coating processes, to regulate the bacterial load per unit area. Finally, dynamically regulating the metabolic activity of *A*. *muciniphila* and tracking its distribution and clearance pathways in the body are crucial for evaluating and eliminating potential toxic side effects.

## 4 Conclusion

In conclusion, this study provides compelling evidence that *Akk*-HA is an effective dual-function biomaterial capable of resisting bacterial colonization and enhancing bone regeneration in infected environments. Through activation of the PI3K/AKT signalling pathway in macrophages, *Akk*-HA stimulates IL-10 production, thereby supporting the osteogenic differentiation of PDLCs. *In vivo* studies further confirmed its therapeutic potential in a murine model of infected periodontal bone defects. These findings support the clinical translation of probiotic-modified bone substitutes as a promising strategy for the treatment of complex bone infections.

## CRediT authorship contribution statement

**Shiyuan Song:** Writing – review & editing, Writing – original draft, Software, Methodology, Investigation, Formal analysis, Data curation, Conceptualization. **Wen Zhang:** Writing – review & editing, Validation, Methodology. **Hongmei Zhuang:** Methodology, Investigation. **Wei Wei:** Methodology, Investigation. **Shuyu Cheng:** Visualization, Methodology. **Dan Qiao:** Writing – review & editing, Investigation. **Yin Xiao:** Writing – review & editing, Supervision, Conceptualization. **Yangheng Zhang:** Writing – review & editing, Supervision, Project administration, Funding acquisition, Conceptualization. **Fuhua Yan:** Supervision, Project administration, Funding acquisition, Conceptualization.

## Ethics approval and consent to participate

Experimental animal protocols were approved by the Animal Ethics Committee of Nanjing Agricultural University (PZ2023042). All animal experiments were authorized by the Animal Ethics Committee and were conducted according to the National Institutes of Health Guide for the Care and Use of Laboratory Animals.

## Declaration of competing interest

The authors declare no conflicts of interest and have all approved this submission.
