# Supplementary material for: Dual functional properties of a probiotic biofilm-decorated bone substitute to combat infection and promote osteoimmunomodulation
Source: Bioact Mater. 2025 Nov 18;57:457–73. doi: 10.1016/j.bioactmat.2025.11.019 (PMC12666132; doi:10.1016/j.bioactmat.2025.11.019)
Supplement: Multimedia component 1 [file mmc1.docx]

Supporting Information

**Dual Functional Properties of a Probiotic Biofilm-Decorated Bone Substitute to Combat Infection and Promote Osteoimmunomodulation**

**This file includes:**

Supplementary Materials and Methods

Fig. S1 to S7

Tab. S1 to S4

**Supplementary Materials and Methods**

**Bacterial culture**

*A. muciniphila* (CICC 24917, Beijing, China) was grown anaerobically in brain heart infusion (BHI; HOPEBIO, Qingdao, China) agar supplemented with 0.4% commercial hog gastric mucin (Sigma‒Aldrich, St. Louis, MO, USA) and 0.05% L-cysteine (Sangon, Shanghai, China). Then, *A. muciniphila* was cultivated in BHI supplemented with 0.2% commercial hog gastric mucin and 0.05% L-cysteine in 15-mL Hungate anaerobic tubes, under anaerobic conditions (80% N₂, 10% H₂, 10% CO₂) at 37 °C.

*F. nucleatum*, *P. gingivalis*, and *S. aureus* were obtained from the Guangdong Microbial Culture Collection Center (Guangzhou, China). *F. nucleatum* and *P. gingivalis* were initially revived on anaerobic blood agar plates supplemented with 5% defibrillated sheep blood and incubated in an anaerobic chamber containing a gas mixture of 10% CO_2_, 10% H_2_, and 80% N_2_. Both strains were subsequently cultured and maintained in Brain Heart Infusion (BHI) liquid medium supplemented with 0.5% yeast extract (Oxoid, Hampshire, UK), 1 μg/mL vitamin K1 (QDRS, Qingdao, China), and 5 μg/mL hemin (Sigma‒Aldrich, St. Louis, MO, USA). Moreover, *S. aureus* was propagated in standard Luria–Bertani (LB) liquid medium under aerobic conditions.

**Bacterial qPCR**

Total bacterial genomic DNA was extracted using a commercial DNA isolation kit (Total DNA Isolation Kit, Vazyme, China). Bacterial suspensions with an optical density of 1 at 600 nm (OD600 = 1) were subjected to tenfold serial dilutions to generate a five-point standard curve. qPCR was performed using species-specific 16S rRNA primers (synthesized by GenScript Biotech, China; primer sequences are provided in Tab. S1) on a 7300 Real-Time PCR System (Applied Biosystems, USA). The obtained cycle threshold (Ct) values were correlated with colony-forming unit (CFU) counts determined by plate counting to construct a standard curve, thereby enabling accurate quantification of bacteria.

**Characterization of *Akk*-HA**

The static contact angles of the samples were measured using a contact angle goniometer (Biolin Scientific, Gothenburg, Sweden). For each measurement, the powder was formed into sheets and carefully deposited on the sample surface with a 2-μL deionized water droplet. After a 5-second equilibrium period, readings were taken, and an image of the outline was captured using an ultra-high-speed camera at 3009 frames per second.

The nanomechanical properties of *Akk*-HA were systematically characterized using a HYSITRON TI 980 nanoindenter (Bruker, Germany). Multiple measurements were performed at different locations on the sample surface under a constant applied load of 200 μN to ensure representative data collection. The resulting mechanical parameters, including the average maximum loading, Young's modulus, and nanoindentation hardness of the biofilm, were determined through statistical analysis of these repeated measurements.

For the crystal violet assay, *A. muciniphila* with an OD600 of 1.0 was diluted to approximately OD600 = 0.1 and cultured with HA for 72 h to form a biofilm. After pasteurization, nonadherent bacteria were removed by washing the biofilm three times with PBS. At predetermined time points, the biofilm was then fixed with methanol for 15 min and stained with 0.01% (w/v) crystal violet for 20 min. The samples were air-dried at room temperature, and images were acquired for documentation.

For the biofilm degradation assay, enzyme solutions were prepared in Tris-HCl buffer with a pH of 7.0 at the following concentrations: 20 μg/mL protease (Vazyme, Nanjing, China), 5 μg/mL amylase (Solarbio, Beijing, China), and 5 μg/mL lipase (Solarbio, Beijing, China). They were added individually or in combination to biofilm-containing wells, followed by static anaerobic incubation at 37°C for 24 h. Following treatment, the biofilm biomass was quantified using crystal violet staining. Then, the stained biofilms were solubilized in 95% anhydrous ethanol, and the absorbance was measured by a microplate reader at 590 nm to determine the relative biofilm biomass. Untreated biofilm served as the negative control. The degradation percentage was calculated using the following formula:

Percentage of degradation = (C−T)/C×100

where:

- *C* = average absorbance of the untreated biofilm (CON)
- *T* = average absorbance of the treated biofilm

**Cell culture**

RAW264.7 cells were provided by Stem Cell Bank, Chinese Academy of Sciences, and were cultured in high-glucose DMEM (Gibco, CA, USA) supplemented with 10% (v/v) foetal bovine serum (FBS; Gibco, CA, USA) and 100 IU/mL penicillin‒streptomycin (P/S; Corning, NY, USA). Every 1 to 2 days, the cells were passaged without trypsin digestion. Human periodontal ligament cells (PDLCs) and murine bone marrow mesenchymal stem cells (BMSCs) were purchased from Zhongqiao Xinzhou Biotechnology Co., Ltd. (Shanghai, China) at passage 2 and were cultured in low-glucose DMEM (Gibco, CA, USA) supplemented with 10% foetal bovine serum and 100 IU/mL penicillin‒streptomycin. The cell culture medium was changed every three days, and when the cells reached 80–90% confluence, the cells were dissociated with 0.05% trypsin-ethylene diamine tetraacetic acid (EDTA) (Gibco, CA, USA). In this study, PDLCs and BMSCs from passages 4 to 6 were utilized.

**Cell viability assay**

Following overnight incubation at 37°C in a 5% CO_2_ humidified atmosphere, RAW264.7 and PDLCs were treated with *Akk*-HA at concentrations of 0, 100, 500, and 1000 ng/mL for 24 h. Cell viability was assessed by both Cell Counting Kit-8 (CCK-8; Vazyme, Nanjing, China) and Calcein/PI Cell Viability and Cytotoxicity Assay Kit (Beyotime, Shanghai, China) staining according to the manufacturers' protocols.

To evaluate the cytotoxicity of the conditioned medium, PDLCs and BMSCs were exposed to macrophage-conditioned osteogenic induction medium (OCM) for 24 h, 48 h, or 72 h. Cell viability was measured at each time point by CCK-8 assay.

**Establishment of animal models and flap surgery**

After inhalation anaesthesia, 4–0 silk ligatures were tied around the bilateral maxillary second molars. In the blank group, the ligature wires were removed immediately after ligation. From the second day of ligation, suspensions of *P. gingivalis* and *F. nucleatum* were applied to the palatal side of the second molar every other day. After ligation for 14 days, the silk ligatures were removed, followed by periodontal flap surgery on the palatal area of the second molar. 5% GelMA 30 hydrogel (EFL, Suzhou, China) with rapid degradability was employed as a transient carrier [1]. The hydrogel was loaded with *Akk*-HA or HA and subjected to in vitro UV curing to create an elastic filler, thus enabling precise packing into the periodontal bone defects of mice [2, 3]. The incisions were made from the mesial surface of the first molar to the distal surface of the third molar on the palatal aspect of the maxilla, and full-thickness flaps were elevated. Two microlitres of either hydrogel alone (CON group) or hydrogel containing 100 μg of material (*Akk*-HA or HA) was implanted into the bone defect area on the palatal aspect of the maxillary second molar (size ≈ 0.5×0.2×0.1 mm³). Then, the wound was fixed using tissue glue (3M™, St. Paul, MN, USA).

**Histological analysis**

Following fixation and decalcification, maxillary specimens underwent dehydration, clearing, and paraffin embedding. Serial sections (4 μm thick) were cut along the mesial-distal plane and stained with haematoxylin & eosin (H&E) and Masson's trichrome (Servicebio, Wuhan, China) for histological assessment. The sections were subsequently mounted and scanned using a light microscope with a panoramic MIDI viewer (3DHISTECH Ltd. EU, Budapest, Hungary). In addition to the maxillae, liver, kidney, and spleen tissues were also stained with H&E following the aforementioned protocol; however, decalcification was not required for these organs.

**Immunohistological staining**

Immunohistological staining was performed to evaluate the levels of inflammation in gingival tissue through a series of procedures. Following dewaxing, paraffin sections underwent antigen retrieval in citric acid buffer and were subsequently blocked with 3% bovine serum albumin (BSA) for 30 min at room temperature.

For immunohistochemistry staining, sections were incubated with diluted antibodies against OPG at 4°C overnight. The sections were subsequently treated with a horseradish peroxidase-conjugated goat anti-rabbit secondary antibody for 30 minutes at room temperature, and DAB was used as the chromogen. The sections were restained using haematoxylin, and images were captured using a digital biopsy scanner (3DHISTECH Ltd. EU, Budapest, Hungary).

For immunofluorescence analysis, sections were triple-stained with antibodies against F4/80, CD206, and CD86 overnight at 4°C, followed by incubation with a mixture of secondary antibodies: Alexa Fluor® 488-conjugated goat anti-mouse IgG, Cy5-conjugated donkey anti-rabbit IgG, and Cy3-conjugated donkey anti-goat IgG for one hour. For the IL-10 and IL-6, sections were incubated with antibodies targeting IL-10 and IL-6 at 4°C overnight, and then with Alexa Fluor® 488-conjugated goat anti-rabbit IgG for one hour. All sections were finally counterstained with 4',6-diamidino-2-phenylindole (DAPI) for 10 min at room temperature to visualize nuclei. Sections were visualized under a light microscope (Nikon, Tokyo, Japan).

**Supplementary Figures**

**
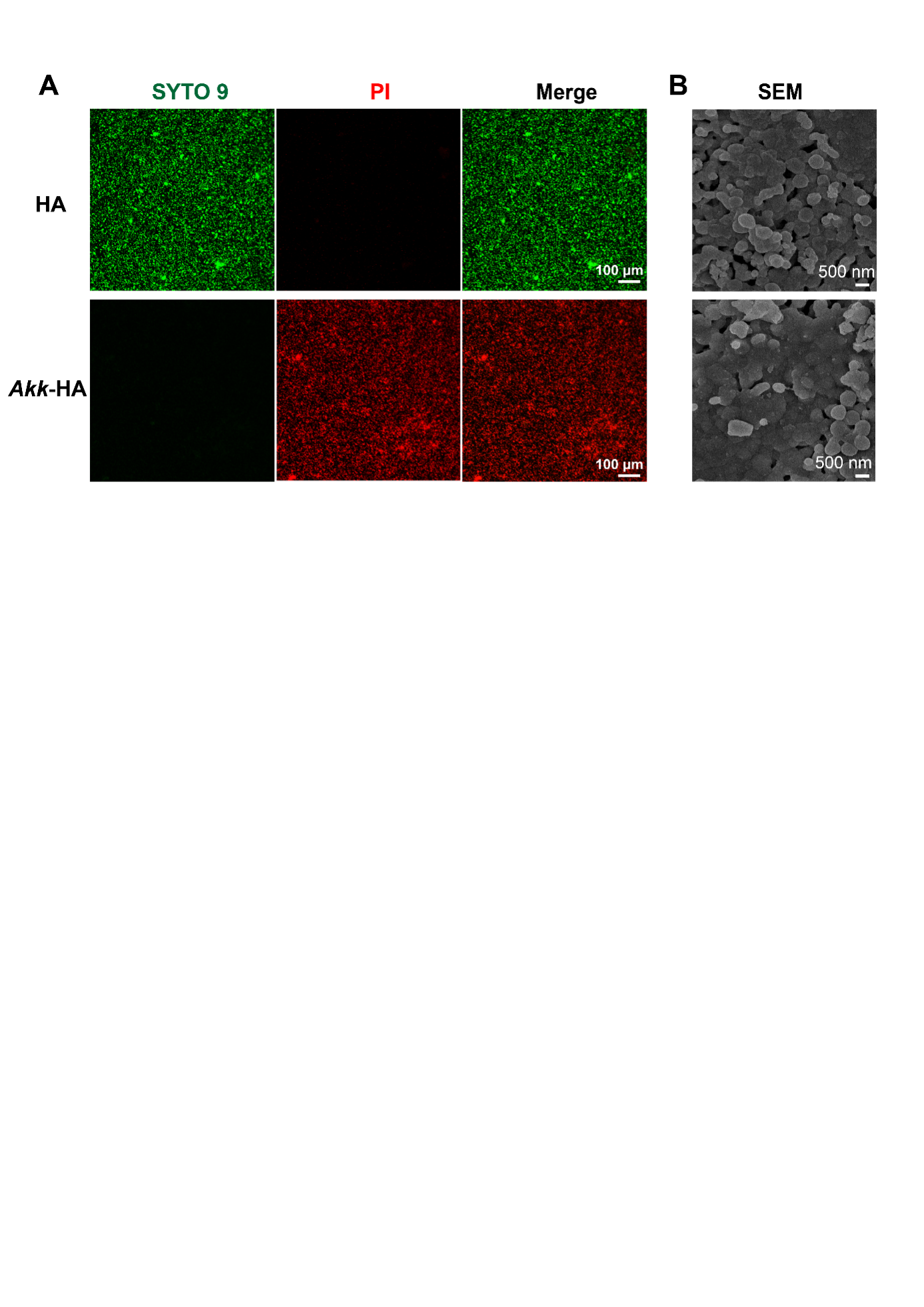
**

**Fig. S1.** Anti-adhesion performance of *Akk*-HA against *S. aureus.* (A) Representative images of SYTO 9/PI staining; red fluorescence indicates *A. muciniphila,* and green fluorescence indicates *S. aureus*. (B) Representative SEM images.


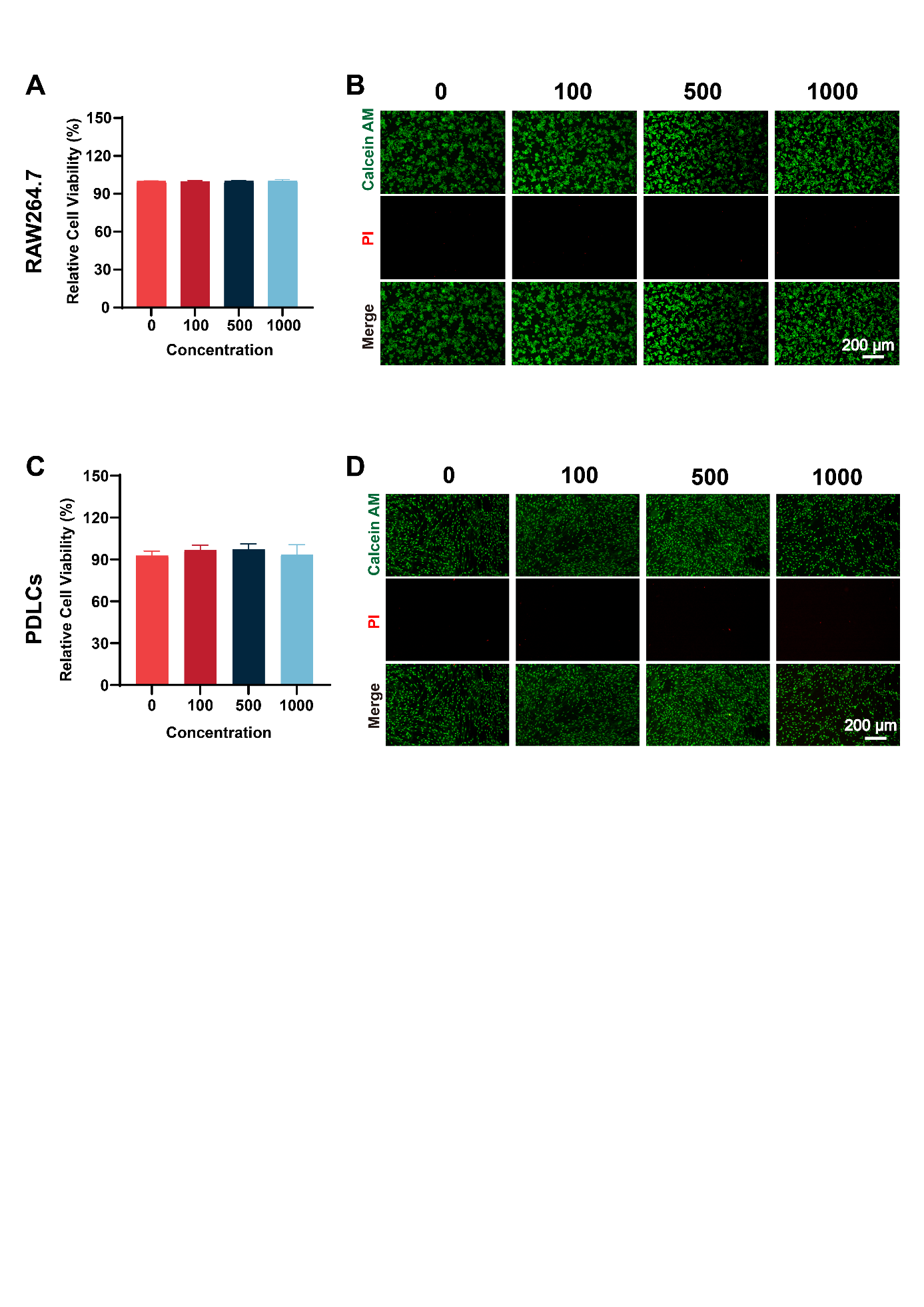


**Fig. S2.** Effects of *Akk*-HA at different concentrations on cell viability and cytotoxicity. (A, C) Viability of macrophages and PDLCs assessed by CCK-8 assay (n = 4). (B, D) Cytotoxicity in macrophages and PDLCs measured by calcein AM/PI staining (n = 4).

**
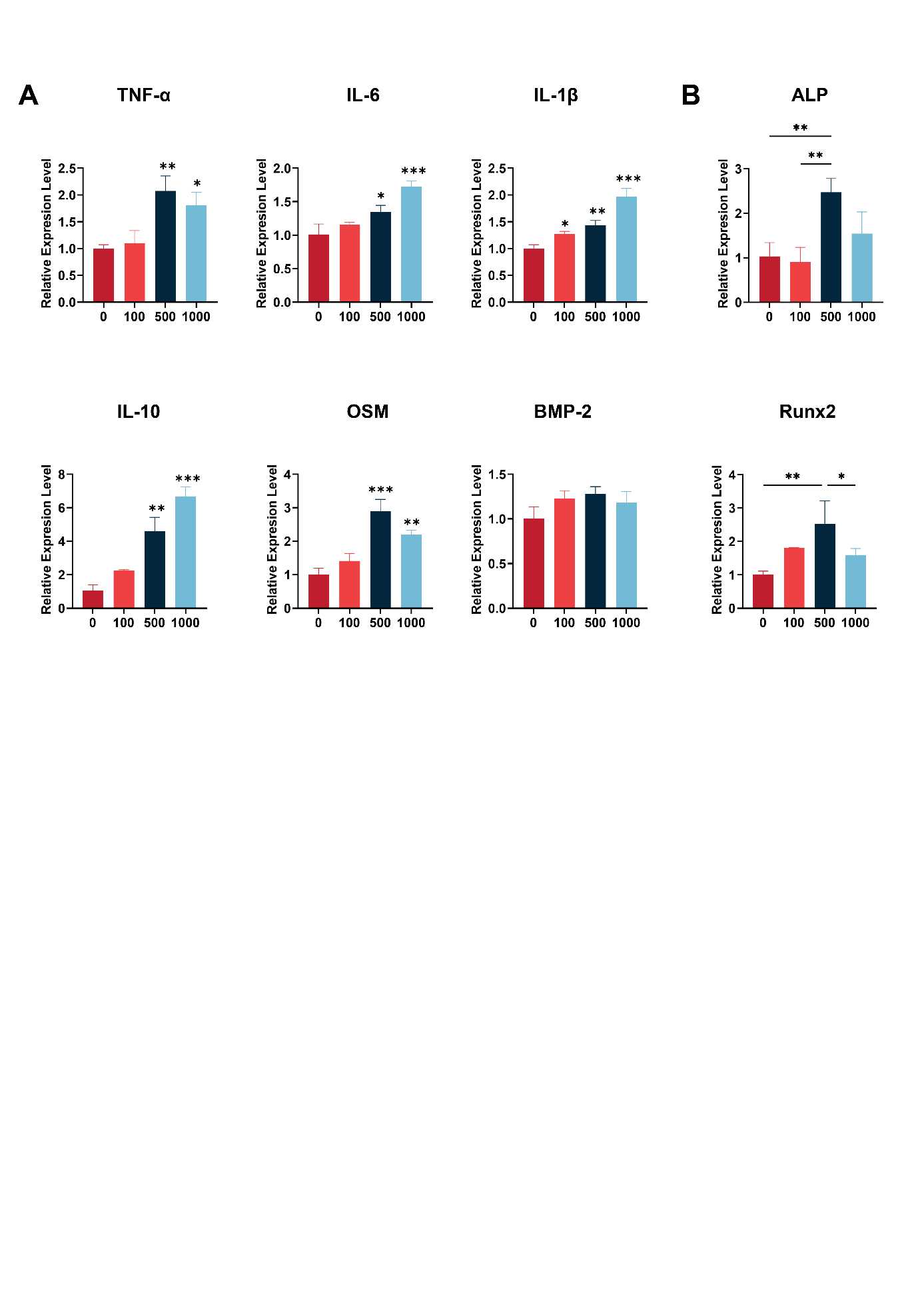
**

**Fig. S3.** Dose-dependent effects of *Akk*-HA on macrophages and PDLCs. (A) Relative mRNA expression levels of inflammatory and regenerative cytokines in macrophages (n = 3). (B) Relative mRNA expression levels of osteogenic genes in PDLCs (n = 3). *P< 0.05, **P< 0.01, and ***P< 0.001.


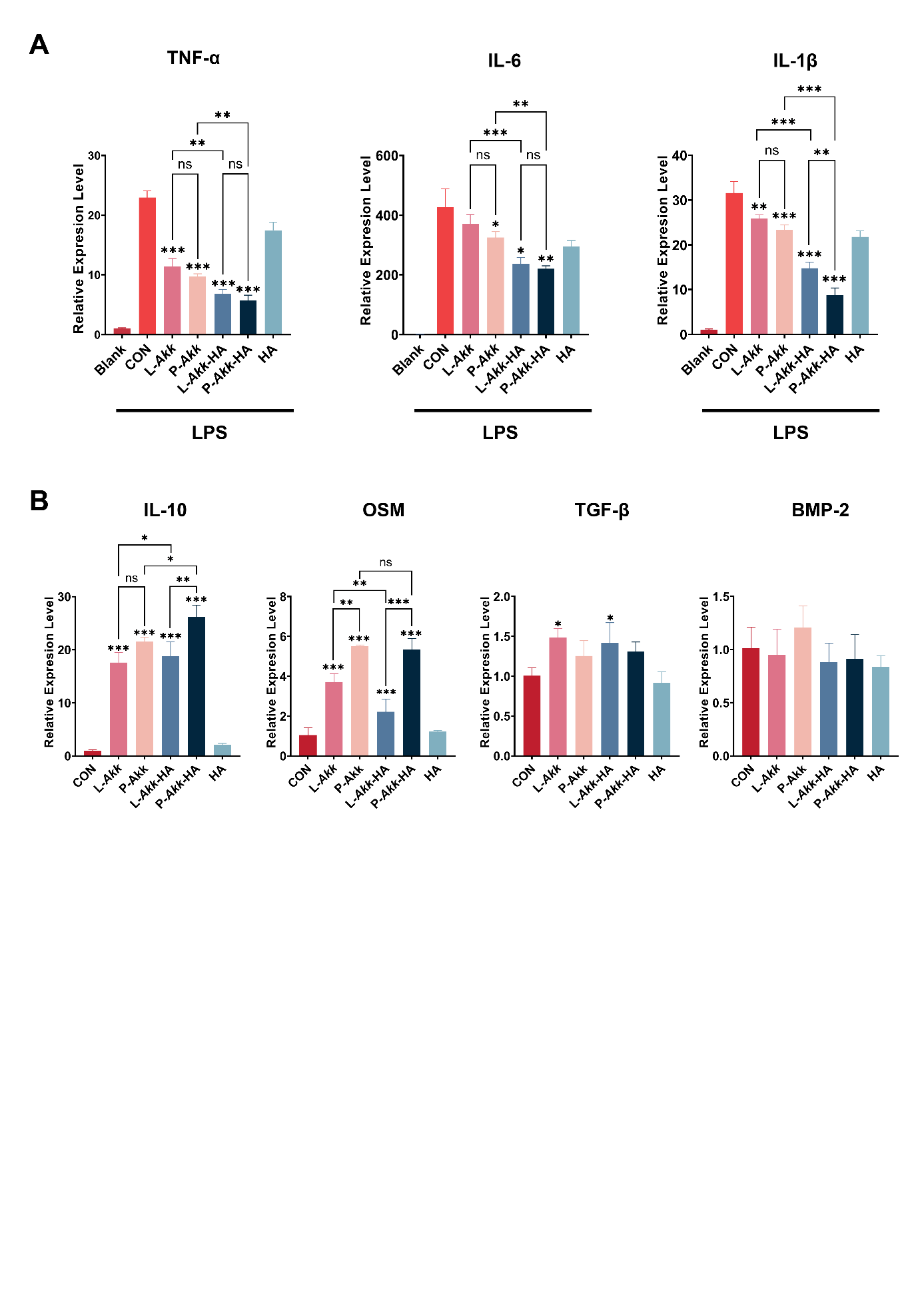


**Fig. S4.** Regulatory effects of L-*Akk,* P*-Akk,* L-*Akk*-HA and P-*Akk*-HA on macrophages. (A) Relative mRNA expression levels of inflammatory cytokines under inflammatory conditions (n = 3). (B) Relative mRNA expression levels of regenerative cytokines (n = 3). *P< 0.05, **P < 0.01, and ***P < 0.001.

**
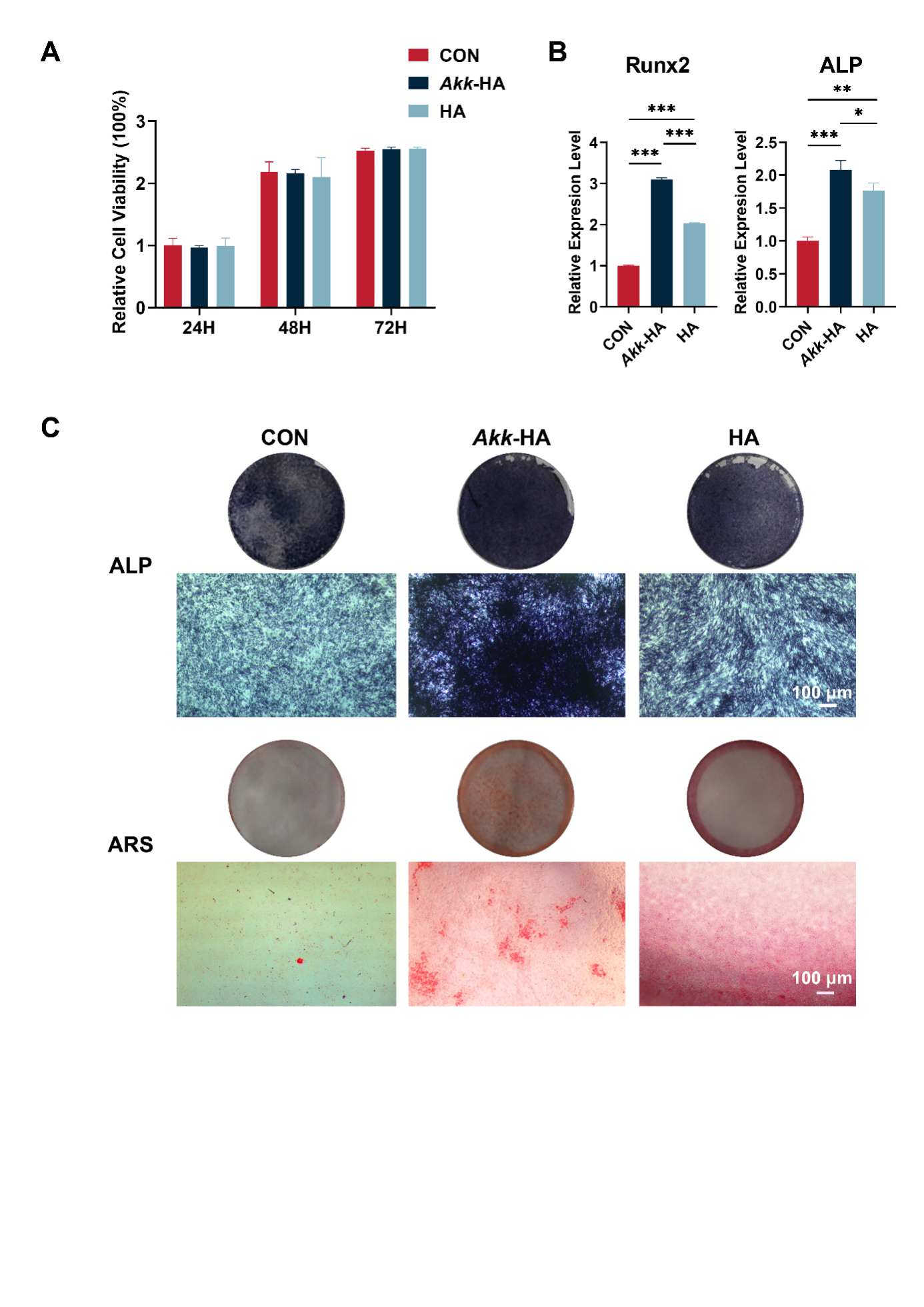
**

**Fig. S5.** Effects of *Akk*-HA-treated macrophage culture supernatant on the osteogenic differentiation of BMSCs. (A) Viability of BMSCs treated with different OCM samples for 72 h (n = 3). (B) Relative mRNA expression levels of osteogenic genes in BMSCs treated with different OCM samples (n = 3). (C) Representative images of ALP staining after 7 days and ARS staining after 21 days in BMSCs cultured in different OCM samples. *P< 0.05, **P < 0.01, and ***P < 0.001.


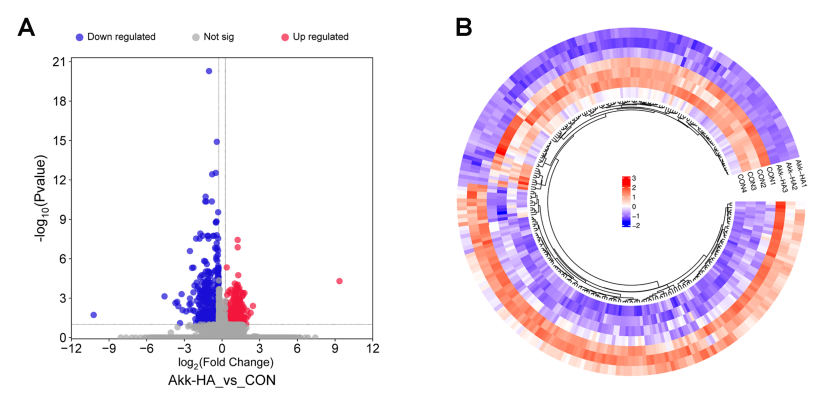


**Fig. S6.** Transcriptomic profile of macrophages treated with Akk-HA. (A) Volcano plot of differentially expressed genes (DEGs). Red and blue dots represent significantly up-regulated and down-regulated genes, respectively. (B) Circular clustering heatmap of the DEGs across different sample groups.


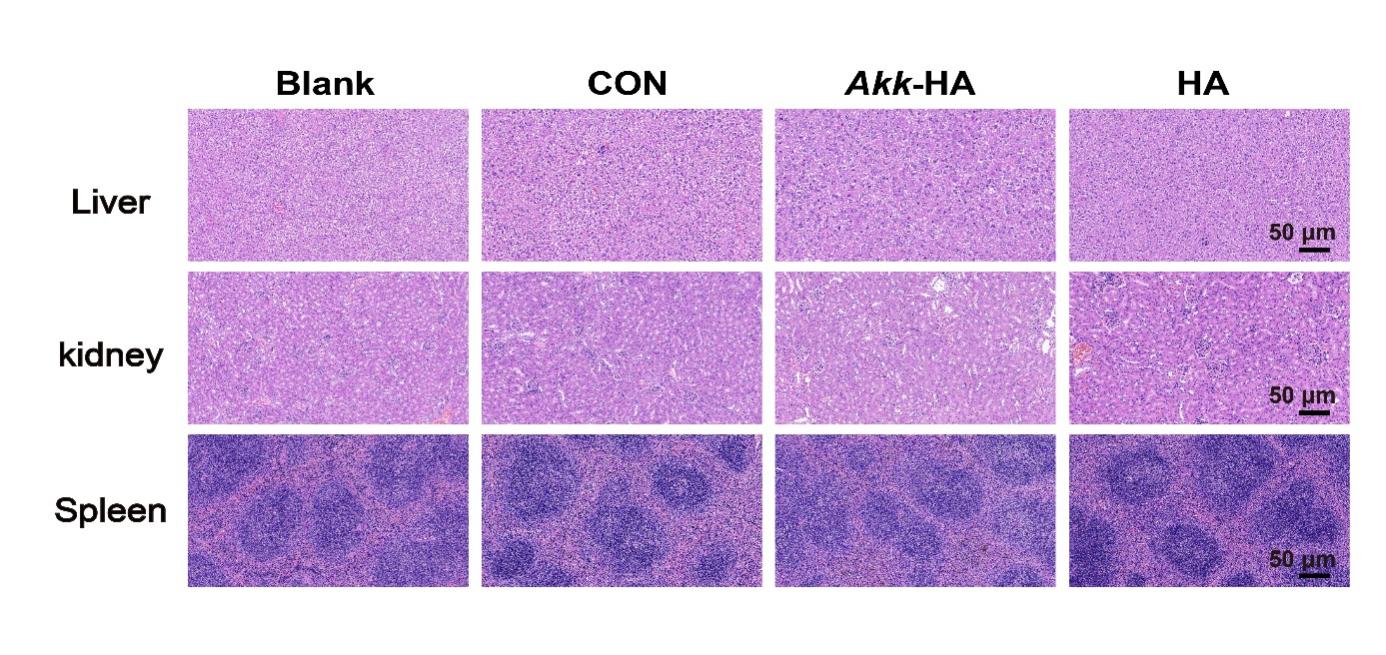


**Fig. S7.**  H&E staining of liver, kidney, and spleen samples.

**Supplementary Tables**

**Tab. S1.** Bacterial primer sequences

| Gene | Forward Primer sequences (5'-3') | Reverse Primer sequences (5'-3') |
| --- | --- | --- |
| *A.muciniphila* | CAGCACGTGAAGGTGGGGAC | CCTTGCGGTTGGCTTCAGAT |
| *P. gingivalis* | CTTGACTTCAGTGGCGGCAG | AGGGAAGACGGTTTTCACCA |
| *F.nucleatum* | CGCAGAAGGTGAAAGTCCTGTAT | TGGTCCTCACTGATTCACACAGA |

**Tab. S2.** RAW264.7 cell primer sequences

| Gene | Forward Primer sequences (5'-3') | Reverse Primer sequences (5'-3') |
| --- | --- | --- |
| TNF-α | AACTCCAGGCGGTGCCTAT | TGCCACAAGCAGGAATGAGA |
| IL-6 | AGTTGCCTTCTTGGGACTGA | TCCACGATTTCCCAGAGAAC |
| IL-1β | AAGGAGAACCAAGCAACGACAAAA | TGGGGAACTCTGCAGACTCAAACT |
| IL-10 | GCCAGAGCCACATGCTCCTA | GATAAGGCTTGGCAACCCAAGTAA |
| OSM | CCCGGCACAATATCCTCGG | TCTGGTGTTGTAGTGGACCGT |
| TGF-β | CTTCAGCCTCCACAGAGAAGAACT | TGTGTCCAGGCTCCAAATATAG |
| BMP-2 | TGAGGATTAGCAGGTCTTTGC | GCTGTTTGTGTTTGGCTTGA |
| GAPDH | AGGTCGGTGTGAACGGATTTG | TGTAGACCATGTAGTTGAGGTCA |

**Tab. S3.** BMSCs primer sequences

| Gene | Forward Primer sequences (5'-3') | Reverse Primer sequences (5'-3') |
| --- | --- | --- |
| Runx2 | AACTTGCTAACGTGAATGGTC | TAGCCCACTGAAGAAACTTGG |
| ALP | ACCACCACGAGAGTGAACCA | CGTTGTCTGAGTACCAGTCCC |
| GAPDH | AGGTCGGTGTGAACGGATTTG | TGTAGACCATGTAGTTGAGGTCA |

**Tab. S4.** PDLCs primer sequences

| Gene | Forward Primer sequences (5'-3') | Reverse Primer sequences (5'-3') |
| --- | --- | --- |
| Runx2 | CAGACAGAAGCTTGATGACTCTAA | CGGGACACCTACTCTCATACT |
| ALP | CACGCCCTTTGCTTTATCT | TCTGAGCCTCCTCTTTTCCT |
| OCN | GATGTGGTCAGCCAACTCGT | GGCAGCGAGGTAGTGAAGAG |
| COL-1 | CTTTGGAGCCAGCTGGA | GTGGGCTTCCTGGTGA |
| GAPDH | TCAGCAATGCCTCCTGCAC | TCTGGGTGGCAGTGATGGC |

**References**

[1] He J, Sun Y, Gao Q, et al. Gelatin Methacryloyl Hydrogel, from Standardization, Performance, to Biomedical Application [J]. Adv Healthc Mater, 2023, 12(23): e2300395. <https://dx.doi.org/10.1002/adhm.202300395>.

[2] Ma W, Lyu H, Pandya M, et al. Successful Application of a Galanin-Coated Scaffold for Periodontal Regeneration [J]. J Dent Res, 2021, 100(10): 1144-52. <https://dx.doi.org/10.1177/00220345211028852>.

[3] Francis M, Gopinathan G, Salapatas A, et al. SETD1 and NF-κB Regulate Periodontal Inflammation through H3K4 Trimethylation [J]. J Dent Res, 2020, 99(13): 1486-93. <https://dx.doi.org/10.1177/0022034520939029>.
